# Biogeography from a food matrix: a temporal distribution map of *Apis mellifera* mitochondrial DNA lineages across Italy, obtained from honey samples

**DOI:** 10.1038/s41598-026-43936-4

**Published:** 2026-03-12

**Authors:** Valeria Taurisano, Anisa Ribani, Maria Letizia Calabri, Giuseppina Schiavo, Kate Elise Nelson Johnson, Valerio Joe Utzeri, Samuele Bovo, Francesca Bertolini, Luca Fontanesi

**Affiliations:** Animal and Food Genomics Group, Division of Animal Sciences, Department of Agricultural and Food Sciences, University of Bologna, Viale Giuseppe Fanin 46, Bologna, 40127 Italy

**Keywords:** Ecology, Ecology, Evolution, Genetics, Molecular biology, Zoology

## Abstract

The genetic integrity of European *Apis mellifera* subspecies has been increasingly threatened. Mitochondrial DNA (mtDNA) analysis is widely used to investigate honey bee lineage distribution. Honey represents a valuable source of environmental DNA (eDNA), containing traces of all the organisms involved in its production, including the honey bees, and can therefore be used to obtain insights on honey bee population genetics. In this study, DNA was extracted from 4292 honey samples produced in all regions of the Italian Peninsula plus Sardinia and Sicily, from 1985 to 2023, and used to provide a temporal snapshot of the distribution of the main *A. mellifera* mtDNA lineages. The C lineage was the most frequent mtDNA throughout Italy, except in Sicily where the A lineage was predominant. MtDNA from the A and M lineages were present in all Italian regions. The frequency of the A mtDNA lineage has increased in many Italian regions over the last years, following a climatic gradient over the Peninsula. Beekeeping practices that used non-native genetic stocks, with the subsequent potential combination of genetic adaptation of honey bees carrying the A lineage to climatic conditions, may explain the observed changes over years in the Italian *A. mellifera* population genetic structure. This is the first temporal biogeographic study obtained from eDNA extracted from a food matrix, namely honey.

**Supplementary Information:**

The online version contains supplementary material available at 10.1038/s41598-026-43936-4.

## Introduction

The most important managed pollinator species, *Apis mellifera* Linnaeus, 1758 (the Western honey bee), is a highly polytypic insect species that includes more than 30 subspecies. These subspecies are characterised by their morphometric features and original geographic distribution, which includes Europe, Africa and Western Asia^1–4^. DNA information, mainly derived from variability at the mitochondrial DNA (mtDNA) level, has subsequently been added to complement morphometric and geographic information and to help construct phylogenetic relationships between *A. mellifera* subspecies^5–8^. These various sources of information partially agree on the definition of five main *A. mellifera* evolutionary lineages^1,3–8^: lineage A, the African lineage; lineage C, of South-Eastern European honey bees; lineage M, of Northern and Western European honey bees; lineage O, of Middle-East honey bees; and the proposed lineage Y, of Arabian and Eastern African honey bees. Italy, including the Peninsula and the main islands of Sicily and Sardinia, is geographically located at the intersection of the natural ranges of four *A. mellifera* subspecies from different evolutionary lineages^1,9–11^: *A. m. ligustica*, *A. m. siciliana*, *A. m. mellifera* and *A. m. carnica*. *Apis mellifera ligustica*, part of the C lineage and native to the Italian Peninsula, predominantly carries the C1 mitotype but has also been reported to carry M7 mitotypes, although the classification of this haplotype requires further refinement^10,12^. *Apis mellifera siciliana*, native to Sicily, is characterised by mitotypes of the A lineage^10,13^. *Apis mellifera mellifera*, belonging to the M lineage and carrying M mitotypes, has been historically found in limited areas of the Western Alps^10^. *Apis mellifera carnica*, also from the C lineage, includes natural populations in northeastern Italy near the Austrian and Slovenian borders, mainly associated with the C2 mitotype^10,14,15^.

Over the last three to four decades, various beekeeping activities and practices, including global commercial queen trade, crossbreeding programs between different subspecies to create improved lines and transhumance across long distances, have greatly contributed to modifying the natural geographic distribution of honey bee populations, thereby affecting the genetic integrity of many European *A. mellifera* subspecies^16–21^. The rising admixture rate between non-autochthonous honey bee subspecies or lines and autochthonous subspecies or ecotypes has raised significant concerns for the conservation of honey bee genetic resources^20–23^. The high level of admixture could lead to the loss of locally adapted genetic characteristics and associated traits, which are regarded as crucial for the sustainable practice of beekeeping and pollination services in various agroecological environments and agricultural production systems^23–28^.

Increased awareness of these issues has facilitated the establishment of conservation programs for autochthonous honey bee subspecies in several European regions^11,12,29–33^. Some of these initiatives have resulted in local regulations or laws, such as those issued in Italy by two administrative regions (Emilia-Romagna and Lazio regions^34,35)^. These conservation programs have been based on monitoring activities aimed at assessing the genetic integrity of the targeted subspecies^11,12,16,20,21,29–33,36^. To achieve this, various studies have used morphometric measurements, mtDNA variability and nuclear genome polymorphisms (such as microsatellites or single nucleotide polymorphisms), either alone or in combination, to obtain a genetic picture of *A. mellifera* populations in specific geographic regions^11,12,16,19–21,29–33,36–42^. However, these pictures are mainly static, capturing information at a single time point determined by the year of sampling of the analysed honey bees, and therefore provide limited information on the trends and changes over time. A few studies based on historical reconstruction from document analysis^43^ or morphometric information from honey bee wing venation^44^ have attempted to reconstruct and track the temporal changes in admixture level and hybridization between different honey bee subspecies across large geographic regions. Other studies have used museum samples to track changes in the genetic diversity of *A. mellifera* over time, either in specific geographic regions^45–47^ or in a large extended area that underwent a sparse sampling approach^48^. These changes have been attributed to selective pressure from the ectoparasite *Varroa destructor*, modifications in beekeeping practices or natural gene flow^45–48^. These studies compared mtDNA and/or nuclear genomic information from pinned dried museum specimens and contemporary honey bees, covering a time period ranging from more than a century to several decades^45–48^. The time distribution was even, based on the availability of past collections^45–48^. However, in order to inform conservation programs for *A. mellifera* genetic resources and establish timely policies, continuous sampling and analysis over a specific timeframe are necessary to track any recent trends and changes in genetic diversity in this managed pollinator species.

Honey contains DNA traces from all organisms that directly or indirectly contributed to its production or are part of the environment and production niche from which this food matrix has been obtained^49–52^. Therefore, as honey is produced by thousands of honey bees forming a colony, traces of their DNA are also present within the honey matrix. Recently, we used these traces to develop diagnostic methods for detecting lineage specific mtDNA haplotypes that can help authenticate the entomological origin of the honey^53,54^. By using assays that can differentiate between the major mtDNA lineages (A, C and M), we have also created an initial distribution map of these honey bee mtDNA lineages covering the entire Italy, based on samples collected in 2018^55^. Commercial honey is typically obtained from multiple colonies or even multiple apiaries, which means that different mtDNA lineages can be identified within the same honey sample. This produces different mtDNA lineage patterns (also indicated as different mtDNA lineage profiles) from the amplification of the DNA extracted from various honey samples: simple mtDNA lineage patterns characterised by only one of the three lineages (exclusively with the presence of the mtDNA A-derived amplified fragment, or the mtDNA C-derived amplified fragment, or the mtDNA M-derived amplified fragment); or complex mtDNA lineage patterns characterised by various combinations of more than one of these three mtDNA lineages (patterns with two lineages: A and C, or A and M; and patterns with all three lineages: A, C and M)^53,55^. This information can be valuable for estimating the spread and prevalence of honey bee lineages^42,55^.

In this study, we monitored the distribution of the main *A. mellifera* mtDNA lineages (A, C and M) throughout Italy over a six-year period. This objective was achieved by analysing DNA from 4,150 honey samples produced across the entire Italian Peninsula, as well as the two main islands, Sardinia and Sicily, between 2018 and 2023. When considering the year of production, these samples were produced by a total of 2,985 different beekeepers (referred to as unique samples). This means that some samples were obtained from the same beekeepers, providing an indirect evaluation of repeated sampling, even if honey was produced from different apiaries and periods of the same year. Additionally, some other samples produced earlier dating back to the 1980s were included (n. 142; grouped into three separate time windows spanning from 1986 to 2017). The results may help to improve understanding of the short-term population genetic changes occurring in *A. mellifera* across the Italian Peninsula and its two major Mediterranean islands, positioned at the crossroads of the geographical distribution of all main European honey bee mtDNA lineages.

## Results

### Overall frequencies of honey samples with different mtDNA lineage patterns in Italy

**Fig. 1:**
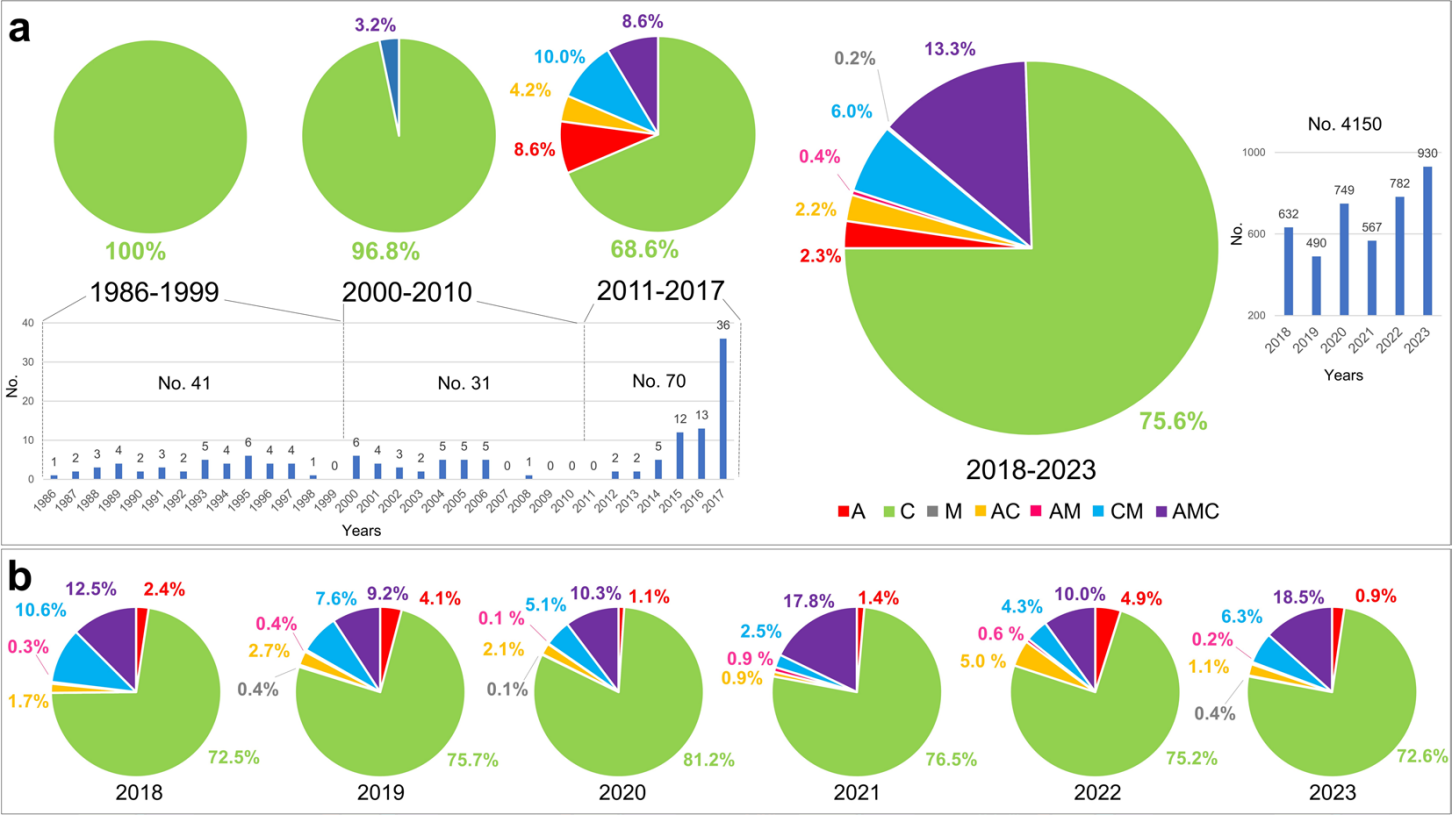
Overall frequencies of the analysed Italian honey samples showing various mtDNA lineage patterns. **(a)** Numbers and frequencies of honey samples produced in the 2018–2023 period, with various lineage patterns, compared to the frequencies of samples produced in 1986–1999, 2000–2009 and 2010–2017. **(b)** Frequencies of the honey samples produced in 2018, 2019, 2020, 2021, 2022, and 2023.

As the typically available honey is produced from more than one colony, and therefore reflects the genetic contribution of more than one queen, the results of the *A. mellifera* mtDNA amplification can show one, two (with all possible combinations) or all three mtDNA (A, C and M) lineages for each sample constituting various mtDNA lineage patterns or profiles, depending on the honey bee population frequencies of the corresponding lineages in a particular production area or apiary. Figure 1 summarises the frequency of the analysed honey samples (calculated from a total of 4,150 samples) with different mtDNA lineage combinations produced in Italy over a six-year period (2018–2023), both overall and divided by year. Honey samples showing only lineage C were consistently the most frequent, representing 75.6% of all samples across the six-year period, and ranging from 72.5% in 2018 to 81.2% in 2020. The C lineage was also the only one detected in most honey samples produced between 1986 and 2017 (Fig. 1). Among honey samples produced between 1986 and 2009, only a single sample, produced in 2001 showed all three mtDNA lineages (A, C and M), whereas all other samples contained only the C lineage. Honey samples showing only the C lineage were also the most frequent (68.6%) among those produced from 2010 to 2017.

The second and third most frequent mtDNA profiles in the period 2018–2023 considering all Italian samples were those with all three mtDNA lineages (A, C and M) and two lineages (C and M), respectively. Their six-year frequencies were 13.3% (ranging from 9.2% in 2019 to 18.5% in 2023) and 6.0% (ranging from 2.5% in 2021 to 10.6% in 2018), respectively.

Similar frequencies are obtained when considering only the 2,985 unique samples, randomly chosen among the samples derived from the same beekeeper within each year (Supplementary Table S1).

### Frequencies of honey-derived mtDNA lineage patterns in Italian regions and macro-regions

**Fig. 2:**
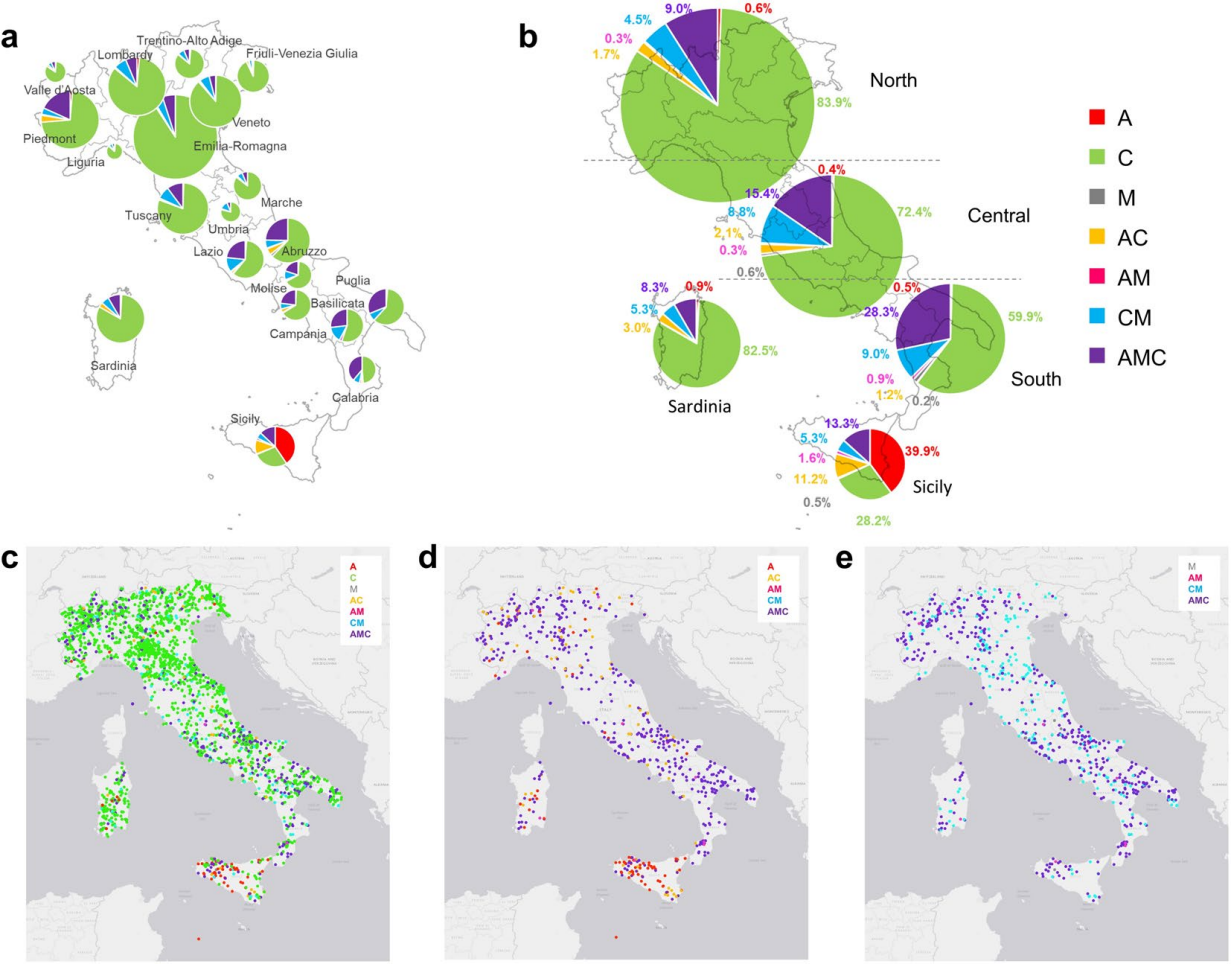
Frequency and distribution maps, divided by Italian regions and macro-regions, of the analysed honey samples across a six-year production period (2018–2023) which resulted to have the indicated mtDNA lineage patterns. **(a)** Frequencies of the honey mtDNA lineage patterns divided by administrative regions and **(b)** macro-regions (North, Central, South, Sicily and Sardinia). Distribution of all analysed honey samples with information on the mtDNA lineage patterns with dots in the maps with the same colours of the legend, **(c)** when considering all honey mtDNA lineage patterns, **(d)** only patterns with A lineage or **(e)** M lineage. The size of the pie charts reported for each region is proportional with the total number of analysed samples produced in the corresponding regions.

Figure 2 reports the frequency of the honey samples produced between 2018 and 2023 showing various mtDNA lineage profiles, divided among the 20 Italian administrative regions and summarised by geographic macro-regions (North, Central, South and the two major islands, Sardinia and Sicily), as well as the spatial distribution of all analysed honey samples during the same period. Density maps of the distribution of all analysed honey samples for each lineage are represented in Supplementary Figure S1. Supplementary Table S1 shows the frequencies across the six-year period and each individual year, divided by administrative regions of production. From these summaries, several geographical patterns and regional differences emerged. The regions with the highest percentage of honey samples produced over the 2018–2023 period with exclusively the C lineage were Friuli-Venezia Giulia (92.3%), Emilia-Romagna (90.5%) and Veneto (88.1%), all located in the North of Italy. Three regions - Emilia-Romagna in northern Italy and Marche and Umbria in central Italy – showed the presence of the C lineage in 100% of honey samples (that means that samples, even if some of them also had other mtDNA lineages, they all contained the C lineage) produced over the six-year period. All other administrative regions, except Sicily, had frequencies of honey samples containing the C lineage above 96% (for details see Supplementary Table S1). Sicily stood out from the rest of Italy, showing the highest frequency of honey samples with only the A lineage (39.9%) or profiles containing the A lineage (66.5%), consistent with the diffusion of the *A. mellifera siciliana* subspecies, which is characterised by the A mtDNA lineage. Calabria showed the second-highest frequency of honey samples with profiles containing the A lineage (43.3%), although none of the samples collected in this region contained only this lineage. The regions with the highest frequency of honey samples containing the M lineage in their profiles were Calabria (48.9%) and Basilicata (44.7%), both located in the South of Italy. However, this lineage alone was not observed in any of the samples from these two regions and only in a few samples from other regions (a total of only 7 samples out of 4,150 analysed).

The frequencies observed when combining regions into macro-regions (North, Central and South of Italy, and the two separated islands), provided a more general geographic overview of the honey produced from 2018 to 2023 (Fig. 2). The North of Italy and Sardinia showed very similar honey mtDNA lineage frequencies. When considering the three macro-regions of the Italian Peninsula, there was an increase in honey samples with the A and M lineages moving from North (A = 11.6%; M = 13.8%), to the Central (A = 18.3%; M = 25.1%) and the South (A = 30.9%; M = 38.4%) of Italy, with a corresponding decrease in honey having the C mtDNA lineage only (North = 83.9%; Central = 72.4%; South = 59.9%).

### Latitudinal distribution of mtDNA lineages patterns across Italy and over years

**Fig. 3:**
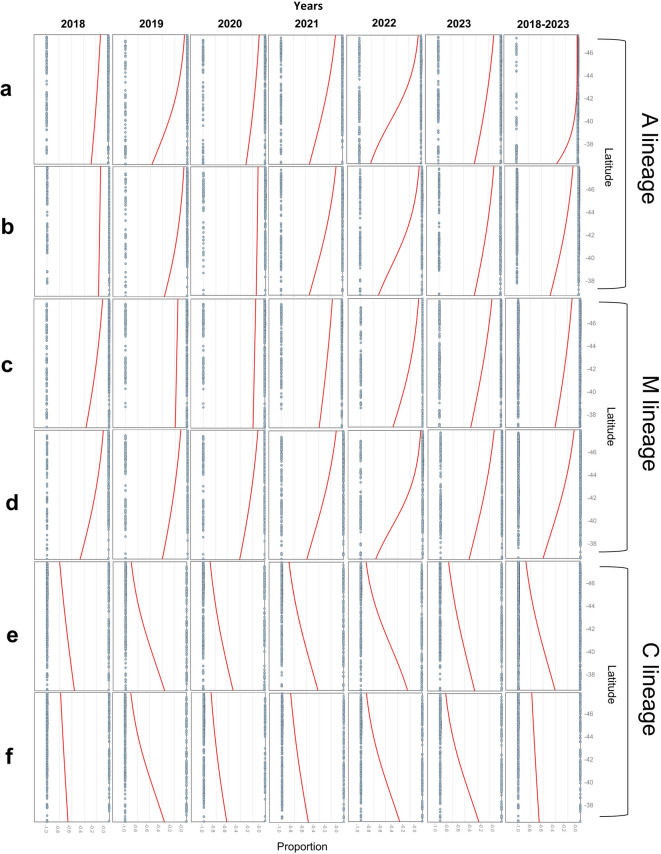
Logistic regression plots showing relationships between latitudinal coordinates and the distribution of honey samples with different mtDNA lineage patterns for each year from 2018 to 2023 and merging all data obtained in the six-year period (2018–2023). **(a)** Distribution of honey samples that had in their patterns the A lineage, including peninsular and Sicilian samples. **(b)** The same as above including peninsular samples only. **(c)** Distribution of honey samples that had in their patterns the M lineage, including peninsular and Sicilian samples. **(d)** The same as above including peninsular samples only. **(e)** Distribution of honey samples that had in their patterns only the C lineage, including peninsular and Sicilian samples. **(f)** The same as above including peninsular samples only.

Again, when considering all honey samples from 2018 to 2023, these trends are further supported by the results of logistic regression analyses based on the latitudinal distribution of the various honey mtDNA profiles (Fig. 3 and Supplementary Table S2). These results indicate that the latitude for the Italian Peninsula (excluding Sicily and Sardinia) is a significant predictor of honey samples with the A lineage or M lineage (that increased moving from North to South) and, in turn for the reverse trend, of honey samples containing only the C lineage (that decreased moving from North to South). Additionally, the significance of the analysis for the A lineage that included Sicily increased, reflecting the high concentration of honey samples with the A lineage produced on this island (Fig. 3 and Supplementary Table S2). No effect was evident when including Sardinia, as their honey lineage profile frequencies are more similar to those of Northern and Central Italy (Supplementary Table S3). Similar results were obtained in the corresponding logistic regression analyses for the honey samples containing only the C lineage, but with opposite frequency trends (Fig. 3 and Supplementary Table S2). For the M lineage, including Sicily (and possibly Sardinia), there was usually a decrease in significance due to the similar frequencies of honey samples with this mtDNA lineage in this island compared to other macro-regions of Italy (Fig. 3; Supplementary Table S2 and S3).

Then, we evaluated whether these latitudinal trends were consistent across all analysed years of this period (2018–2023) or if there were temporal differences. The logistic regression described above was therefore run separately for each year of production (Fig. 3; Supplementary Tables S2). The latitude for the Italian Peninsula (excluding Sicily and Sardinia) was not a significant predictor of honey samples with A mitotypes in 2018 and 2020, but it was significant in 2019, 2021, 2022 and 2023. This pattern contributed substantially to the overall trend observed across 2018–2023. When we included Sicilian honey samples along with those from the Peninsula, the latitude was consistently a more significant predictor of honey samples with the A lineage in all years, confirming the high concentration of honey with the A lineage in Sicily throughout the years (Fig. 3 and Supplementary Tables S2 and S3). For honey samples with the M lineage produced in the Peninsula, latitude was a significant predictor in 2019, 2020, 2021, 2022 and 2023. In 2018 it was close to the nominal threshold for significance (*p* = 0.053). When including Sicily in this analysis for honey samples with the M lineage, significance was reached in 2018 (*p* = 0.021) as well as in 2019, 2021, 2022 and 2023 (with much more significant values), but not in 2020. However, when significant, the Chi-square value of the model was always lower than that obtained in the analysis conducted only with the Peninsula data, indicating that the frequency of Sicilian honey samples with the M lineage was not a defining characteristic of this island. Furthermore, for honey samples containing only the C lineage (Peninsula alone or Peninsula + Sicily), latitude was a significant predictor in the opposite direction in all years, except for the Peninsula-only analysis in 2018 (Fig. 3 and Supplementary Tables S2 and S3).

### Longitudinal distribution of mtDNA lineage patterns across the north of Italy and over years

**Fig. 4:**
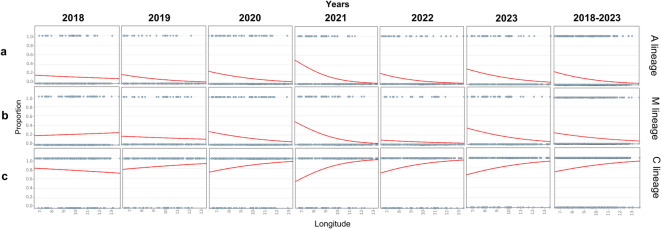
Logistic regression plots showing relationships between longitudinal coordinates from Northern Italy and the distribution of honey samples with different mtDNA lineage patterns for each year from 2018 to 2023 and merging all data obtained in the six-year period (2018–2023). **(a)** Distribution of honey samples that had in their patterns the A lineage. **(b)** Distribution of honey samples that had in their patterns the M lineage **(c)** Distribution of honey samples that had in their patterns only the C lineage.

The longitudinal distribution of mtDNA lineage patterns (honey samples with the A lineage, the M lineage or only the C lineage) was evaluated across Northern Italy, which spans from 6° 37’ E (the westernmost longitude) to 14° 1’ E (the easternmost longitude) for 564 km. The longitudinal analysis was conducted only for this geographic part of Italy because Northern Italy is bordered in the westernmost and easternmost parts by the contact regions of the natural distribution of the *A. m. mellifera* and *A. m. carnica* subspecies, respectively^9–11^. Additionally, Northern Italy spans the largest continuous longitudinal coordinates of this Peninsula, bordered by natural barriers constituted by the Alp arch from the West, North and East and by the Apennine mountains from the South. Initially, all honey samples produced from 2018 to 2023 were analysed together in logistic regression analyses with geographic coordinates as variables. For honey samples with A or M mtDNA haplotypes, longitude was a significant predictor, showing decreasing frequencies from west (highest frequencies) to east (lowest frequencies; Fig. 4 and Supplementary Table S4). In contrast, for honey samples with only the C lineage, longitude was a significant predictor, with increasing frequencies from west (lowest frequencies) to east (highest frequencies), in the opposite direction compared to samples with A and/or M lineages (Fig. 4 and Supplementary Table S4). Next, we examined whether these longitudinal trends remained consistent across all analysed years from 2018 to 2023 or if there were temporal differences (Fig. 4 and Supplementary Table S4). For honey samples with the A lineage, longitude was not a significant predictor for samples produced in 2018, whereas it was highly significant in all other years. Similarly, for honey samples with only the C lineage, longitude was not significant in 2018 and 2019, but significant in all other years. For honey samples with the M lineage, longitude was not a significant predictor in 2018, 2019 and 2022.

### Changes of frequencies of honey samples with different mtDNA lineage patterns across macro-regions over years

**Fig. 5:**
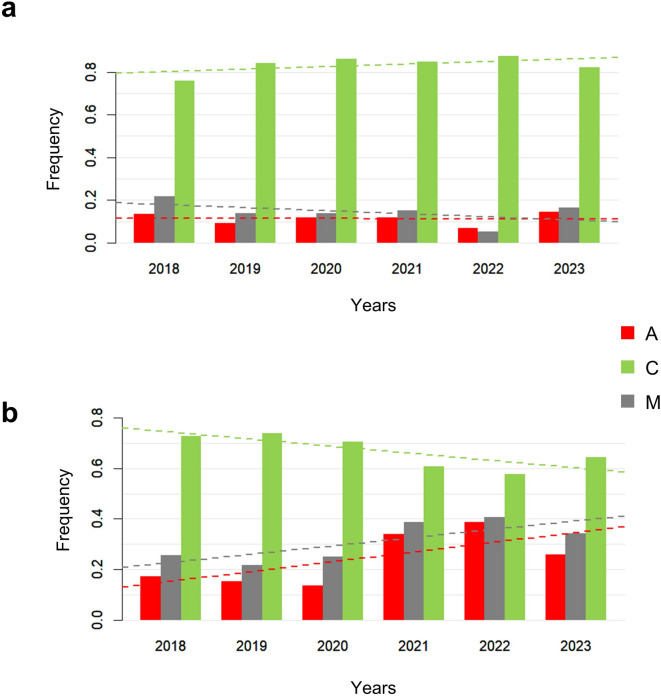
Frequency of honey samples with different lineage combinations (patterns with the A lineage, patterns with the M lineage and with only the C lineages) in various years across macro-regions. **(a)** Northern Italy. **(b)** Central + Southern Italy.

We then tested whether the frequency of mtDNA lineage patterns within macro-regions changed over the years between 2018 and 2023. To minimize potential sampling biases, we only included macro-regions and years with more than 100 honey samples in this analysis. Therefore, we considered only the North of Italy and the Central + South of Italy. Figure 5 shows the frequency of honey samples with different mtDNA lineage patterns (honey with the A lineage, with the M lineage or with only the C lineage) categorized by production year and macro-region. P-values of the logistic regression conducted over years for the various mtDNA lineage profiles are provided in Supplementary Table S5. In Northern Italy, we observed slight but significant trends over time, with decreasing frequencies of honey samples containing the M lineage and increasing frequencies of honey samples containing only the C lineage. The frequency of honey samples with the A lineage remained stable over time. In Central + Southern Italy, we observed significant increasing trends in the frequencies of honey samples with A and M lineages, while honey samples with only the C lineage decreased over time.

### Comparison of results from honey samples produced by the same beekeepers

**Fig. 6:**
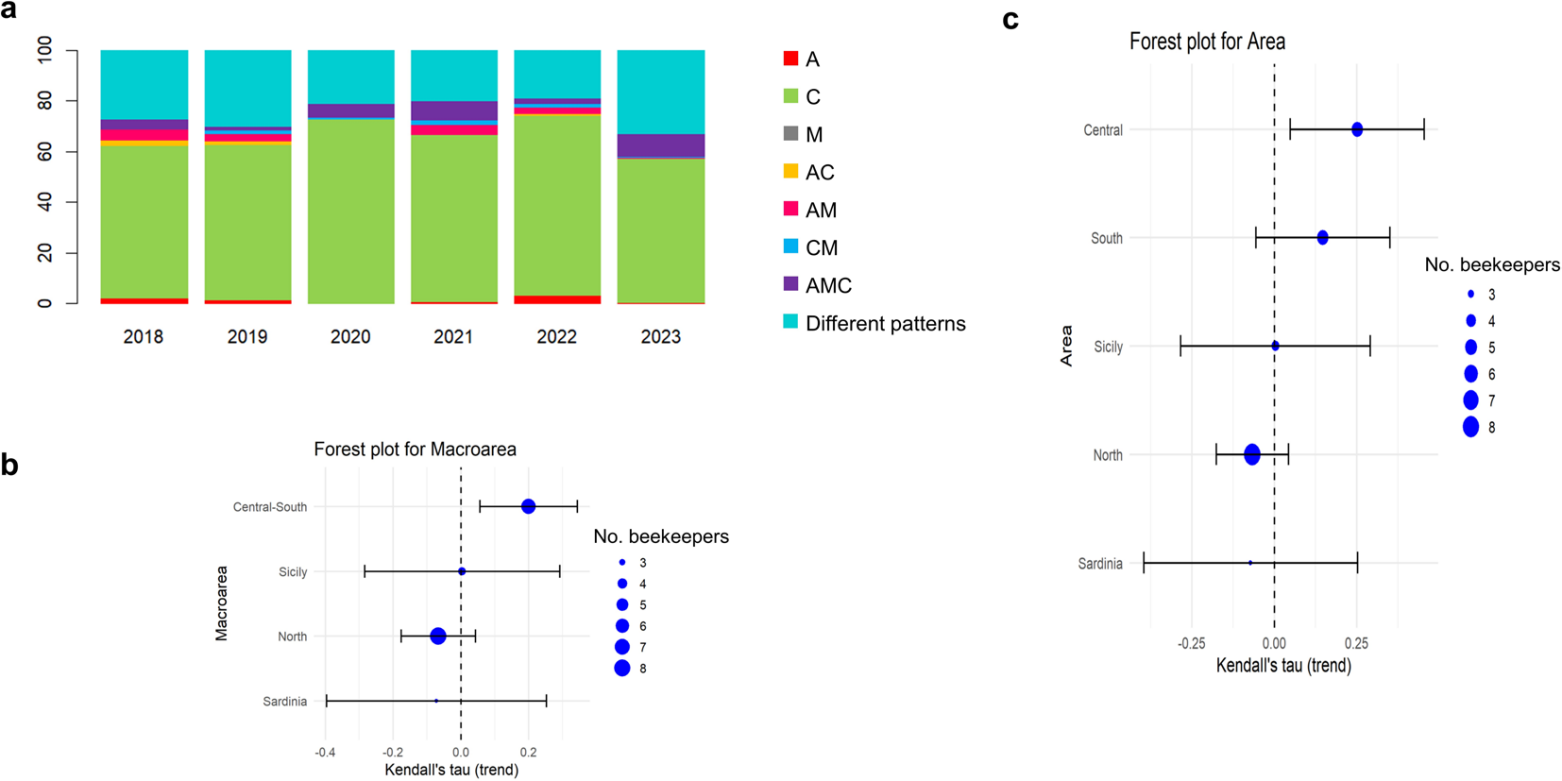
Trends of mtDNA patterns obtained from honey samples produced by the same beekeepers over years. (**a)** Barplots of the frequency of mtDNA lineage profiles for honey samples provided by the same beekeeper within each year of production. The bar for any of the six years represents the proportion of beekeepers who, within the same year, provided more than one honey sample. “Different patterns” indicates the proportion of beekeepers that provided honey samples produced in the same year that did not have the same mtDNA lineage patterns for all samples provided. (**b**,** c)** Forest plots of Kendall’s τ trends across geographical areas. Each dot represents the mean Kendall’s τ estimated for the corresponding group. The macro-area plot in (b) combines the four main geographical areas into broader regions (North, Central–South, and the two Islands. The size of each dot is proportional to the number of beekeepers contributing to that group.

We initially evaluated the consistency of mtDNA profiles for honey samples provided by the same beekeeper within each year of production, indicated as “redundant” samples (see Supplementary Table S6 for the numbers of honey samples). The number of samples ranged from a minimum of two to a maximum of nine honey samples produced in the same year. Figure 6a summarises, by production year, the number of beekeepers who provided honey samples with a consistent mtDNA lineage profile and those who provided samples with different profiles, regardless of the number of redundant samples submitted for the considered year. In all years, the largest number of beekeepers provided samples containing only the C lineage. This pattern was driven primarily by beekeepers who provided two samples for the considered year, with decreasing numbers among those who provided up to nine samples. Over the years, there was a noticeable increase in the number of beekeepers who, in the same production year, provided a greater number of samples showing different profiles. This ranged from 27.1% in 2018 to 32.9% in 2023. Correspondingly, the proportion of beekeepers who provided only samples containing only the C lineage decreased from 60.4% in 2018 to 57.1% in 2023.

We then considered the mtDNA lineage profiles of the honey samples provided by the same beekeepers over multiple years. We only included beekeepers who submitted samples in at least four years between 2018 and 2023. We conducted a meta-analysis using the Mann Kendall test, looking at the number of different lineages provided by each beekeeper over the years, categorized by macro-regions (with Central and Southern Italy analysed either separately or combined). Figure 5 displays the Forest plots of this analysis. The Kendall’s τ values were positive for both Central and Southern Italy, as well as when these two macro-regions were combined. This suggests an increasing trend over time in beekeepers submitting honey samples with multiple lineages, indicating increased diversity in the honey samples and implying that their apiaries became more heterogeneous in terms of mtDNA lineages. This trend was primarily driven by beekeepers from Central Italy. The Kendall’s τ values for Northern Italy and Sardinia were slightly negative, while for Sicily, it was neutral but with wide standard errors.

## Discussion

In this population genetic study, we used a food matrix, honey, as a source of genetic information from the organisms that produced it, namely honey bees. By analysing geolocalized honey samples produced over the years, we obtained a picture that showed how the population genetic structure of *A. mellifera* changed over time in a large geographic region that included all of Italy. Italy can be geographically distinguished into various parts following the latitudinal profile of its Peninsula (North, Central and South) and its two main Mediterranean islands, Sardinia and Sicily. The longitudinal profile can be obtained from the Northern Italy, which cover more than 500 km from West to East, touching at both extreme ends the regions of the natural diffusion of *A. m. mellifera* and *A. m. carnica* subspecies, respectively^9–11^. Therefore, the resulting picture could also refer to macro-region levels as well as region levels considering the 20 administrative regions of Italy. This regional level is particularly relevant given that some administrative regions (Emilia-Romagna and Lazio) have enacted laws to preserve the genetic integrity of the native honey bee subspecies, *A. m. ligustica*^12,34,35^.

Honey bee DNA contained in honey is usually highly degraded^52–55^. For this reason, only short DNA fragments can be amplified and analysed, making it possible to capture limited sequence information. In our case, this was sufficient to distinguish the three main *A. mellifera* mtDNA lineages (A, C and M), using a short region of the COI-COII intergenic spacer. This region includes 30-end bases of the tRNALeu gene and the non-coding P and Q regions, which can be easily differentiated using a simple three fragments length-based assay, that separates A, C and M amplified fragments^53^. Consistent with the specific characteristics of the analysed honey, samples frequently showed complex patterns determined by the co-presence of two or three mtDNA lineages, indicating that they derived from apiaries where colonies had queens with different lineages. Since the assay was qualitative rather than quantitative, in cases where more than one mtDNA lineage was detected per sample, the results indicated mtDNA heterogeneity in the *A. mellifera* population managed by the beekeeper who provided the sample. This approach may overestimate the overall frequency of mtDNA lineages, particularly those that are less represented, and specifically the A and M lineages. To evaluate this potential issue, we can use the information on mtDNA haplotype frequencies that we recently obtained by analysig a total of 1143 honey bees sampled in the Emilia-Romagna region from 2020 to 2022^12^. This information can be compared with the results of the honey-derived mtDNA lineage frequencies reported in the current study for the same years (a total of 576 honey samples, from different beekeepers than those who provided honey bees in the previous study). The frequencies of honey bees carrying A and M mtDNA haplotypes were 1.3% and 1.1%, respectively, accounting for a total of 2.4% of the analysed honey bees^12^. The frequencies of honey samples with mtDNA lineage profiles including either A or M lineages were 3.5% (20 out of 576 samples) and 5.4% (31 out of 576 samples), respectively. When considering all honey samples showing either A and M lineages together, the frequency was 5.9% (34 out of 576 samples), similar to the 5.4% reported above, as most of the honey samples containing the M lineage also contained the A lineage. This may indicate that honey samples could have a 2.46 time more effective capacity to capture low frequency mtDNA lineages. Nevertheless, it is notable that comparable frequencies of the A and M lineages were obtained using the two methods, namely direct analysis of individual honey bees^12^ and indirect estimation from honey samples (this study).

The higher sensitivity of honey eDNA in capturing low frequency mtDNA lineages may result in higher informativity when monitoring the distribution and frequency trends of mtDNA lineages over years and geographic areas, compared to studies that analyse individual honey bees directly for the same purpose. This also makes the use of honey eDNA more cost-effective, especially when a large number of samples need to be characterised to achieve the geographic and temporal coverage that was obtained in the current study. We can estimate that each honey sample may provide genetic information from approximately 10 to 30 colonies, on average, substantially enlarging the population genetic information derived from each sample.

Using this strategy, we successfully analysed a total of 4292 honey samples, including 142 old samples produced from 1986 to 2017 and an additional 4150 samples produced in the six-year time window spanning from 2018 to 2023. To our knowledge, the samples produced in the 1980’s are the oldest ones that were successfully analysed to retrieve genetic information on the honey bees that produced them. Other archived older honey samples could potentially be the source of sufficiently intact DNA to retrieve short mtDNA haplotypes. Despite the limited geographic distribution of old honey samples (Supplementary Table S7), it was interesting to note that most of the samples produced before 2010 had only the C lineage. This homogeneous genetic information changed over time, as demonstrated by the results obtained from honey produced from 2010 to 2023, with more details in the 2018–2023 time window.

Our initial study based on honey samples produced only in 2018^55^ provided updated information on the geographic distribution of *A. mellifera* mtDNA lineages in Italy. In comparison to the geographic distribution of honey bee mtDNA lineages in Italy provided by Franck et al.^10^ in 2000, our studies highlighted some discrepancies. The study of Franck et al.^10^ was based on mtDNA analyses from approx. 660 individual honey bees sampled in a few apiaries from 17 sites, with 14 of those sites located on the Peninsula. We already noted that the frequency of the M mtDNA lineage reported by Franck et al.^10^ was approx. 50%, which was much higher than what we observed in our honey samples from 2018 to 2023 (Fig. 1). In our current study, only around 20% of honey samples showed the M lineage, which can also be considered an overestimation of the frequency of this lineage in the Italian honey bee population. Furthermore, only 0.17% of honey samples produced from 2018 to 2023 had exclusively the M lineage, suggesting a low relative frequency of this lineage in Italy. This reduced frequency of the M lineage in the Italian honey bee population has also been recently reported by Yadró Garcia et al.^56^ who analysed 216 drones collected in 2022 from different apiaries across the Italian Peninsula. These authors reported a frequency of 23.6% for the M lineage, which appears to be higher than the estimation that could be deduced from the current study and from what we reported in Taurisano et al.^12^ from honey bees sampled in 2020–2022 in the Emilia-Romagna region. Additionally, Franck et al.^10^ identified the A lineage only in two Sicilian sites, where all honey bees carried mtDNA of this lineage. In contrast, our updated distribution map shows the presence of the A lineage spread throughout Italy (Figs. 1 and 2), confirming what we initially reported from honey samples produced in 2018^55^. This has also been confirmed by Yadró Garcia et al.^56^ who reported a frequency of 4.3% for this lineage in honey bees sampled in Italy, as mentioned above.

If we examine the results obtained from honey produced between 2018 and 2023 as a whole, there is a clear latitudinal continental gradient for all three mtDNA lineages. The frequency of honey samples with the A and/or M lineage increased while, conversely, the frequency of honey samples with only the C lineage decreased from North to South. These clines are similar to those reported by Yadró Garcia et al.^56^ with a much lower number of honey bee samples analysed, collected only in 2022. When Sicily is included in this geographical analysis alongside the Peninsula, the trends for the honey samples with A mitotypes and those with only the C lineage (moving in opposite direction) were much more significant. This was due to the high frequency of the A lineage in *A. m. siciliana* populations on the island. However, when we analysed each year separately within this time frame, the trends for the Peninsula were not significant in 2018 and only partially in 2020. This suggests that changes occurred over time based on pronounced waves alternated with some more stable periods, leading to a substantial redefinition of Italian honey bee population genetic structures globally. These different trends could potentially also be due in part to stochastic effects derived from the sampling of the analysed honey. Additionally, when considering macro-regions, it seems that the increased frequency of heterogeneous honey samples in terms of mtDNA lineages observed across the Central and Southern Peninsula predominantly occurred from 2021, after an initial stable period from 2018 to 2020. This latitudinal shift is also supported by the increased frequency over time of honey provided by the same beekeepers with more than one mtDNA lineage.

When we monitored these trends with a longitudinal analysis over Northern Italy, the overall picture for 2018–2023 was consistent with an increased frequency of honey samples with only the C lineage (and conversely, decreased frequencies of honey samples with A and M lineages) going West to East, which was more pronounced between 2020 and 2023. This can be explained by the increased frequency of the C2 mitotype, which is characteristic of the *A. m. carnica* subspecies^15,54^. This is especially evident, when we are closer to the native area of this subspecies (near the Slovenia border), as we also reported when we analysed a few honey samples using a test that can distinguish C1 from C2 mitotypes^54^. In the current study, since we did not differentiate between honey samples with the C lineage into the two main mtDNA haplotypes C1 and C2, this hypothesis can only be indirectly suggested.

Some hypotheses can explain the observed overall picture and trends on the distribution of the three main *A. mellifera* mtDNA lineages in Italy, that are clearly different compared to previously reported pictures^10,55^. The primary factor contributing to these changes may be attributed to beekeeping practices that used non-native genetic stocks. This could potentially lead to a combination of genetic adaptation within the honey bee populations, allowing them to better withstand changing climatic conditions. The distribution of the A lineage follows a decreasing gradient from South to North on the Peninsula, mirroring the temperature gradient, with the South of Italy that experiences a dry and warm climate in contrast to the cooler regions further North. The wide-spread presence of the African mtDNA lineage all over continental Italy points to a primary human-mediated dispersion, mainly determined by the use of hybrid queens derived from non-autochthonous lines, referred to as Buckfast (not fixed for any mtDNA lineage^56–60)^, or the introduction of non-native subspecies, as we have already suggested^55^. The importation of queens from South America, mainly Argentina and Chile, where Africanized honey bees have been identified^61,62^, could have created an additional potential route of introgression into the Italian populations. Another hypothesis could consider the potential expansion or spread of *A. m. siciliana*, which carries the A lineage^56^. The extensive use of non-autochthonous lines has been considered the main force that has been modifying honey bee population genetic structures in many other parts of Europe^16,21,37,39,40,44^. The introduction of lines carrying the A lineage seems to be stable in Northern Italy, where many professional beekeepers may routinely use hybrid queens. The higher increase of A and M lineages in Central and Southern Italy since 2021 could indicate a potential rise in international commercial activity, leading to extensive use of non-native genetic stocks by beekeepers. The commercial spread of *A. m. carnica* in Northern Italy over the last years^63^ may also explain, at least in part, the observed West-East gradient. Statistics on queen-bee trade show an increase in queen trade in Italy from 2019 to 2022, but do not differentiate between internal production and importation^64^. On the other hand, the latitudinal trend over time of African mitotypes could be also partially attributed to a climate driven gradient potentially influenced by the higher adaptation capacity to warm temperatures of colonies with African genetics^65,66^. In this context it will be important to assess the productive and behavioral characteristics of colonies carrying the A lineage compared with those carrying other lineages.

The mtDNA results, however, do not allow us to estimate the extent of introgression at the nuclear genome level. To obtain this information for Italian populations, further research based on whole-genome sequencing or on genotyping large panels of nuclear DNA markers, also proposed on honey eDNA, is required^51,67^.

## Conclusions

To our knowledge, this is the first biogeographic study obtained from eDNA extracted from a food matrix that included a temporal evaluation of genetic information. Honey produced over multiple years has revealed temporal changes in the population genetic structure of the Italian *A. mellifera* populations. When considering mtDNA as a marker of population genetic information, it is evident that the Italian *A. mellifera* populations are becoming heterogeneous, with an increased frequency of African mitotypes showing a latitudinal gradient. The updated map of honey bee mtDNA lineages that we produced indirectly from honey can assist in designing and assessing the effectiveness of conservation policies and actions aimed at preserving the diversity and integrity of honey bee genetic resources in Italy.

## Methods

### Honey samples

A total of 4150 honey samples produced between 2018 and 2023 were collected. These samples were produced in all Italian regions, comprising the Peninsula and the two main islands of Sicily and Sardinia. The number of honey samples for each year and region of production is shown in Supplementary Table 1. Some beekeepers provided more than one sample for the same year of production; therefore, we constructed a list named as “unique” samples per year, where we randomly retained only one sample per beekeeper for each year (Supplementary Table 1). Honey samples produced from the same beekeepers were indicated as “redundant” samples even if they were produced in different periods of the year, from different botanical sources and, potentially only partially from the same colonies and apiaries from which the “unique” samples were obtained. Supplementary Table S6 reports detailed information on the number of samples provided by the same beekeepers over the same year or all six years of production.

Honey samples produced from 2018 to 2023 were geographically positioned in the map of Italy using longitude and latitude coordinates of the localities of the apiaries from which they have been produced. When honey samples were from beekeepers that owned more than one apiary or when the precise apiary information was not available, the geographic coordinates were from the municipality (“Comune”), where the honey was produced.

From different sources, including old collections and beekeepers’ repositories, we also collected other 142 honey samples produced from 1986 to 2017, with a few gaps, in 16 Italian regions. All samples produced from 1986 to 2004 (n. 61) were from the Emilia-Romagna region, which accounted a total of 75 samples produced in the 1986–2017 time-window. To simplify the representation of the results, samples produced in this period were divided into three groups based on the year of production: 1986–1999, no. 31; 2000–2009, no. 31; and 2010–2017, no. 70. Supplementary Table S7 reports details for these samples, including year of production and geographical locations.

### DNA extraction from honey

DNA extraction from honey samples followed the protocol utilised in previous studies^52–54^. This protocol involved initial steps to remove sugars through centrifugation and washing of the pelleted materials, followed by isolation of the DNA contained in the pellets. This latter part of the protocol included resuspending the pellet in 0.5 mL of ultrapure water and adding it to 1 mL of CTAB extraction buffer [2% (w/v) cetyltrimethylammoniumbromide; 1.4 M NaCl; 100 mM Tris-HCl; 20 mM EDTA pH 8], along with 5 µL of RNase A solution (10 mg/mL) and 30 µL of proteinase K (20 mg/mL). This mixture was then incubated at 65 °C for 90 min with gentle mixing. After cooling to room temperature, the tube was centrifuged for 10 min at 16,000 g. A total of 700 µL of the supernatant was transferred to a new tube containing 500 µL of chloroform/isoamyl alcohol (24:1), vortexed for 30 s and then centrifuged at 16,000 g for 15 min at room temperature. The supernatant was moved to a 1.5 mL tube, and the DNA was precipitated with isopropanol and then ethanol/water in a 70:30 v/v ratio, following a standard protocol. The precipitated DNA was rehydrated with 30 µL of sterile H_2_O and stored at − 20 °C until PCR analyses.

### PCR analyses

The amplification of the DNA isolated from honey samples was based on the primer pair utilised by Utzeri et al.^53,55^: forward, 5′-GGCAGAATAAGTGCATTG-3’; reverse, 5′-TTAATATGAATTAAGTGGGRAAW-3′. This primer pair amplifies an *A. mellifera* mtDNA region that can discriminate three major mtDNA lineages: A, C and M. Supplementary Figure S2 reports a schematic representation of the amplified and diagnostic mtDNA region. As positive controls, all amplifications included DNA from three honey bees carrying each one of the three targeted mtDNA lineages, derived from Taurisano et al.^12^. PCR analyses were carried out in a total volume of 14 µL using KAPA HiFi HotStart master mix (Kapa Biosystems, Roche Molecular Systems, Basel, Switzerland) on a 2700 thermal cycler (Life Technologies; Carlsbad, CA, USA). PCR cyclings were the same as those reported by Utzeri et al.^53^. The obtained amplicons were electrophoresed in 4.0% agarose gels in TBE 1X buffer and stained with 1X GelRed nucleic acid gel stain (Biotium Inc., Fremont, CA, USA). Supplementary Figure S3 shows a few examples of electrophoretic patterns obtained with the fragments of the three main mtDNA lineages.

### Data analyses

The frequency distribution of honey samples with different lineage combinations (also indicated as patterns or profiles) was calculated for each region and year of production from 2018 to 2023. The frequency of honey samples with different lineage combinations was also obtained for groups of years, considering the period 2018–2023, and for the samples produced before 2018, the three-time windows described above (1986–1999, 2000–2009, and 2010–2017).

Subsequent biogeographical analyses were conducted following Utzeri et al.^55^ and included only honey samples produced from 2018 to 2023, that were from all Italian regions. Density maps were created using ArcGIS Online (ESRI, https://www.arcgis.com/index.html, accessed on 20th February 2025) utilising the Calculate Density tool. These density maps were based on honey samples containing the targeted mtDNA lineages, either alone or in combination with others, to construct different maps of mtDNA lineage groups.

Logistic regression was utilised to test relationships between the distribution of a particular lineage or combination of mtDNA lineages (patterns or profiles) across geographic coordinates, either latitude or longitude. Latitude positions were analysed across the entire Italian Peninsula, with the option to include or exclude the two major Italian islands, Sardinia and Sicily. Longitude positions were analysed across regions in the North of Italy, including honey samples produced in Piedmont, Valle d’Aosta, Liguria, Lombardy, Trentino-Alto Adige, Veneto, Friuli-Venezia Giulia and Emilia-Romagna regions. In the models, mtDNA lineages were coded as binary variables to account for honey samples with or without a specific mtDNA lineage, including or excluding honey samples with multiple mtDNA lineages.

Logistic regression was also utilised to test changes of mtDNA lineage frequencies (determined by counting honey samples having a specific mtDNA lineage) over years, within macro-regions (defined by dividing Italy in North, Central and South of Italy and the two main islands considered alone, and considering together the Central and South of Italy; Supplementary Table S1). Logistic regression models were implemented with the R *glm* package, with default parameters.

Temporal trends in the dataset were assessed using the Modified Mann–Kendall (MMK) test, a non-parametric approach that quantify the strength and direction of monotonic changes across time series. The MMK test corrects the variance of the original Mann–Kendall statistic for the presence of autocorrelation in short and potentially dependent values, as it is the case for the analysed genetic data. In detail, each beekeeper was codified as a vector of values indicating the genetic diversity of mtDNA lineages for each year. This value could span from 1 to 3, depending on the number of different mtDNA lineages occurring in honey samples provided for a particular year. Only producers that provided honey for at least four years were included in MKK test. The resulting τ values and their corresponding variances were subsequently analysed through a meta-analysis framework using the R package *metafor*^68^. Random-effects meta-analytic mixed models were fitted using the Restricted Maximum Likelihood (REML) estimator and using geographical areas (North, Central, South, Sicily and Sardinia) or macro-areas (North, Central and South considered together, Sicily, and Sardinia) as modifiers and considering a confidence interval of 95%. Forest plots were obtained to visualize the direction and magnitude of the Kendall’s τ from the pooled value of the macro-areas, providing a synthesis of temporal dynamics across them.

## Supplementary Information

Below is the link to the electronic supplementary material.

Supplementary Material 1

## Data Availability

Detailed information on honey samples with the mtDNA profiles has been deposited in Zenodo with DOI: 10.5281/zenodo.18046460.
