# Supplementary material for: Biogeography from a food matrix: a temporal distribution map of Apis mellifera mitochondrial DNA lineages across Italy, obtained from honey samples
Source: Sci Rep. 2026 Mar 12;16:13280. doi: 10.1038/s41598-026-43936-4 (PMC13106849; doi:10.1038/s41598-026-43936-4)

Supplementary Material 1

Biogeography from a food matrix: a temporal distribution map of *Apis mellifera* mitochondrial DNA lineages across Italy, obtained from honey samples

Valeria Taurisano, Anisa Ribani, Maria Letizia Calabri, Giuseppina Schiavo, Kate Elise Nelson Johnson, Valerio Joe Utzeri, Samuele Bovo, Francesca Bertolini and Luca Fontanesi

Supplementary Table S1. Frequencies of honey-derived mtDNA lineage patterns divided by regions and macro-regions considering the group of years from 2018 to 2023 and for single years in this period.

Regions[1]	Years[2]	All samples											Unique samples										
		No. of honey samples[3]	A	C	M	AC	AM	CM	ACM	A*	C*	M*	No. of honey samples[4]	A	C	M	AC	AM	CM	ACM	A*	C*	M*
Piedmont (Piemonte)	2018-2023	388	5	281	0	15	2	14	71	93	381	87	321	4	233	0	13	2	13	56	75	315	71
Valle d'Aosta	2018-2023	90	1	75	0	3	0	5	6	10	89	11	44	1	37	0	2	0	2	2	5	43	4
Liguria	2018-2023	63	1	54	0	0	1	4	3	5	61	8	50	1	43	0	0	1	2	3	5	48	6
Lombardy (Lombardia)	2018-2023	402	3	301	0	10	1	18	69	83	398	88	325	3	243	0	7	1	17	54	65	321	72
Trentino-Alto Adige	2018-2023	145	2	120	0	3	1	10	9	15	142	20	112	2	91	0	3	0	9	7	12	110	16
Veneto	2018-2023	270	1	238	1	2	0	17	11	14	268	29	175	1	148	1	2	0	14	9	12	173	24
Friuli-Venezia Giulia	2018-2023	143	1	132	0	3	1	4	2	7	141	7	81	1	73	0	1	1	4	1	4	79	6
Emilia-Romagna	2018-2023	873	0	790	0	6	0	34	43	49	873	77	473	0	436	0	2	0	13	22	24	473	35
Tuscany (Toscana)	2018-2023	186	1	149	0	2	0	15	19	22	185	34	160	1	127	0	2	0	13	17	20	159	30
Umbria	2018-2023	66	0	52	0	1	0	9	4	5	66	13	56	0	45	0	1	0	7	3	4	56	10
Marche	2018-2023	127	0	109	0	2	0	8	8	10	127	16	103	0	87	0	2	0	6	8	10	103	14
Lazio	2018-2023	170	2	103	1	3	1	21	39	45	166	62	137	2	82	1	1	1	19	31	35	133	52
Abruzzo	2018-2023	157	0	98	3	7	1	9	39	47	153	52	118	0	75	2	7	1	6	27	35	115	36
Molise	2018-2023	86	1	56	0	2	1	9	17	21	84	27	69	1	45	0	1	1	7	14	17	67	22
Campania	2018-2023	130	1	85	1	5	0	8	30	36	128	39	111	1	72	1	5	0	8	24	30	109	33
Puglia	2018-2023	225	1	138	0	0	0	16	70	71	224	86	171	1	109	0	0	0	12	49	50	170	61
Basilicata	2018-2023	123	0	68	0	0	3	19	33	36	120	55	99	0	53	0	0	3	17	26	29	96	46
Calabria	2018-2023	90	0	44	0	2	2	7	35	39	88	44	78	0	37	0	2	2	6	31	35	76	39
Sicily (Sicilia)	2018-2023	188	75	53	1	21	3	10	25	124	109	39	156	64	41	0	18	3	10	20	105	89	33
Sardinia (Sardegna)	2018-2023	228	2	188	0	7	0	12	19	28	226	31	146	1	121	0	5	16	5	14	36	145	35
**All Italy**	**2018-2023**	**4150**	**97**	**3134**	**7**	**94**	**17**	**249**	**552**	**760**	**4029**	**825**	**2985**	**84**	**2198**	**5**	**74**	**32**	**190**	**418**	**608**	**2880**	**645**
Piedmont (Piemonte)	2018	56	0	46	0	0	0	3	7	53	10	10	55	0	45	0	0	0	3	7	7	55	10
Valle d'Aosta	2018	8	0	7	0	0	0	1	0	7	1	1	8	0	7	0	0	0	1	0	0	8	1
Liguria	2018	9	0	8	0	0	0	1	0	8	1	1	9	0	8	0	0	0	1	0	0	9	1
Lombardy (Lombardia)	2018	71	0	50	0	3	0	6	12	62	18	21	68	0	49	0	2	0	6	11	13	68	17
Trentino-Alto Adige	2018	23	1	8	0	3	0	8	3	11	11	14	22	1	8	0	3	0	8	2	6	21	10
Veneto	2018	41	0	28	0	1	0	7	5	33	12	13	40	0	27	0	1	0	7	5	6	40	12
Friuli-Venezia Giulia	2018	22	0	20	0	0	0	2	0	20	2	2	22	0	20	0	0	0	2	0	0	22	2
Emilia-Romagna	2018	101	0	84	0	0	0	7	10	94	17	17	61	0	52	0	0	0	2	7	7	61	9
Tuscany (Toscana)	2018	37	0	29	0	0	0	4	4	33	8	8	36	0	28	0	0	0	4	4	4	36	8
Umbria	2018	15	0	12	0	1	0	1	1	13	2	3	15	0	12	0	1	0	1	1	2	15	2
Marche	2018	22	0	18	0	0	0	0	4	22	4	4	22	0	18	0	0	0	0	4	4	22	4
Lazio	2018	29	1	21	0	0	0	4	3	24	7	7	29	1	21	0	0	0	4	3	4	28	7
Abruzzo	2018	22	0	18	0	0	0	1	3	21	4	4	21	0	17	0	0	0	1	3	3	21	4
Molise	2018	10	0	6	0	1	0	2	1	7	3	4	10	0	6	0	1	0	2	1	2	10	3
Campania	2018	26	0	19	0	0	0	2	5	24	7	7	25	0	18	0	0	0	2	5	5	25	7
Puglia	2018	37	1	25	0	0	0	2	9	34	11	11	34	1	22	0	0	0	2	9	10	33	11
Basilicata	2018	20	0	12	0	0	0	5	3	15	8	8	20	0	12	0	0	0	5	3	3	20	8
Calabria	2018	9	0	5	0	0	0	2	2	7	4	4	9	0	5	0	0	0	2	2	2	9	4
Sicily (Sicilia)	2018	29	12	7	0	0	2	5	3	12	10	8	28	11	7	0	0	2	5	3	16	15	10
Sardinia (Sardegna)	2018	45	0	35	0	2	0	4	4	39	8	10	24	0	20	0	1	0	1	2	3	24	3
**All Italy**	**2018**	**632**	**15**	**458**	**0**	**11**	**2**	**67**	**79**	**537**	**146**	**157**	**558**	**14**	**402**	**0**	**9**	**2**	**59**	**72**	**97**	**542**	**133**
Piedmont (Piemonte)	2019	49	2	38	0	0	1	2	6	9	46	9	46	2	35	0	0	1	2	6	9	43	9
Valle d'Aosta	2019	21	1	18	0	0	0	1	1	2	20	2	8	1	5	0	0	0	1	1	2	7	2
Liguria	2019	7	0	6	0	0	0	0	1	1	7	1	6	0	5	0	0	0	0	1	1	6	1
Lombardy (Lombardia)	2019	50	1	40	0	1	0	5	3	5	49	8	44	1	36	0	0	0	5	2	3	43	7

Trentino-Alto Adige	2019	21	0	20	0	0	0	1	0	0	21	1	14	0	14	0	0	0	0	0	0	14	0
Veneto	2019	25	0	24	0	0	0	0	1	1	25	1	16	0	15	0	0	0	0	1	1	16	1
Friuli-Venezia Giulia	2019	21	0	21	0	0	0	0	0	0	21	0	8	0	8	0	0	0	0	0	0	8	0
Emilia-Romagna	2019	64	0	50	0	0	0	8	6	6	64	14	38	0	32	0	0	0	3	3	3	38	6
Tuscany (Toscana)	2019	24	0	21	0	0	0	0	3	3	24	3	23	0	21	0	0	0	0	2	2	23	2
Umbria	2019	12	0	11	0	0	0	1	0	0	12	1	12	0	11	0	0	0	1	0	0	12	1
Marche	2019	14	0	13	0	0	0	1	0	0	14	1	14	0	13	0	0	0	1	0	0	14	1
Lazio	2019	37	1	29	0	3	0	4	0	4	36	4	25	1	20	0	1	0	3	0	2	24	3
Abruzzo	2019	14	0	10	0	1	1	1	1	3	13	3	14	0	10	0	1	1	1	1	3	13	3
Molise	2019	7	0	6	0	0	0	0	1	1	7	1	7	0	6	0	0	0	0	1	1	7	1
Campania	2019	13	0	7	1	3	0	2	0	3	12	3	13	0	7	1	3	0	2	0	3	12	3
Puglia	2019	27	0	19	0	0	0	4	4	4	27	8	26	0	18	0	0	0	4	4	4	26	8
Basilicata	2019	15	0	10	0	0	0	5	0	0	15	5	12	0	8	0	0	0	4	0	0	12	4
Calabria	2019	12	0	3	0	0	0	0	9	9	12	9	12	0	3	0	0	0	0	9	9	12	9
Sicily (Sicilia)	2019	38	14	12	1	2	0	0	9	25	23	10	31	13	10	0	2	0	0	6	21	18	6
Sardinia (Sardegna)	2019	19	1	13	0	3	0	2	0	4	18	2	19	1	13	0	3	0	2	0	4	18	2
**All Italy**	**2019**	**490**	**20**	**371**	**2**	**13**	**2**	**37**	**45**	**80**	**466**	**86**	**388**	**19**	**290**	**1**	**10**	**2**	**29**	**37**	**68**	**366**	**69**
Piedmont (Piemonte)	2020	87	0	68	0	0	0	0	19	19	87	19	63	0	46	0	0	0	0	17	17	63	17
Valle d’Aosta	2020	25	0	19	0	0	0	3	3	3	25	6	9	0	8	0	0	0	0	1	1	9	1
Liguria	2020	8	0	8	0	0	0	0	0	0	8	0	6	0	6	0	0	0	0	0	0	6	0
Lombardy (Lombardia)	2020	75	0	59	0	0	0	0	16	16	75	16	49	0	40	0	0	0	0	9	9	49	9
Trentino-Alto Adige	2020	24	0	19	0	0	1	0	4	5	23	5	14	0	11	0	0	0	0	3	3	14	3
Veneto	2020	74	0	71	0	0	0	1	2	2	74	3	31	0	29	0	0	0	1	1	1	31	2
Friuli-Venezia Giulia	2020	37	0	36	0	0	0	0	1	1	37	1	14	0	13	0	0	0	0	1	1	14	1
Emilia-Romagna	2020	193	0	170	0	0	0	7	16	16	193	23	103	0	93	0	0	0	3	7	7	103	10
Tuscany (Toscana)	2020	28	0	23	0	0	0	0	5	5	28	5	27	0	22	0	0	0	0	5	5	27	5
Umbria	2020	9	0	7	0	0	0	2	0	0	9	2	9	0	7	0	0	0	2	0	0	9	2
Marche	2020	16	0	16	0	0	0	0	0	0	16	0	16	0	16	0	0	0	0	0	0	16	0
Lazio	2020	21	0	11	0	0	0	10	0	0	21	10	20	0	11	0	0	0	9	0	0	20	9
Abruzzo	2020	15	0	10	1	4	0	0	0	4	14	1	14	0	9	1	4	0	0	0	4	13	1
Molise	2020	16	1	12	0	0	0	2	1	2	15	3	13	1	10	0	0	0	2	0	1	12	2
Campania	2020	19	1	13	0	1	0	4	0	2	18	4	18	1	12	0	1	0	4	0	2	17	4
Puglia	2020	22	0	15	0	0	0	2	5	5	22	7	21	0	14	0	0	0	2	5	5	21	7
Basilicata	2020	14	0	9	0	0	0	5	0	0	14	5	13	0	9	0	0	0	4	0	0	13	4
Calabria	2020	16	0	8	0	1	0	2	5	6	16	7	16	0	8	0	1	0	2	5	6	16	7
Sicily (Sicilia)	2020	29	6	14	0	9	0	0	0	15	23	0	23	6	10	0	7	0	0	0	13	17	0
Sardinia (Sardegna)	2020	21	0	20	0	1	0	0	0	1	21	0	20	0	19	0	1	0	0	0	1	20	0
**All Italy**	**2020**	**749**	**8**	**608**	**1**	**16**	**1**	**38**	**77**	**102**	**739**	**117**	**499**	**8**	**393**	**1**	**14**	**0**	**29**	**54**	**76**	**490**	**84**
Piedmont (Piemonte)	2021	49	0	31	0	0	1	0	17	18	48	18	39	0	27	0	0	1	0	11	39	12	11
Valle d’Aosta	2021	11	0	9	0	0	0	0	2	2	11	2	3	0	3	0	0	0	0	0	3	0	0
Liguria	2021	11	0	9	0	0	1	0	1	2	10	2	10	0	8	0	0	1	0	1	10	2	1
Lombardy (Lombardia)	2021	70	0	48	0	0	1	0	21	22	69	22	57	0	39	0	0	1	0	17	57	18	17
Trentino-Alto Adige	2021	14	1	11	0	0	0	0	2	3	13	2	11	1	8	0	0	0	0	2	10	2	2
Veneto	2021	51	0	50	0	0	0	0	1	1	51	1	24	0	23	0	0	0	0	1	24	1	1
Friuli-Venezia Giulia	2021	26	0	26	0	0	0	0	0	0	26	0	12	0	12	0	0	0	0	0	12	0	0
Emilia-Romagna	2021	120	0	114	0	0	0	5	1	1	120	6	67	0	66	0	0	0	1	0	66	1	1
Tuscany (Toscana)	2021	20	0	17	0	0	0	0	3	3	20	3	18	0	15	0	0	0	0	3	18	3	3
Umbria	2021	5	0	4	0	0	0	1	0	0	5	1	5	0	4	0	0	0	1	0	4	1	1
Marche	2021	23	0	15	0	1	0	6	1	2	23	7	20	0	14	0	1	0	4	1	15	5	6
Lazio	2021	26	0	10	0	0	0	0	16	16	26	16	22	0	9	0	0	0	0	13	22	13	13
Abruzzo	2021	17	0	9	0	0	0	0	8	8	17	8	13	0	8	0	0	0	0	5	13	5	5
Molise	2021	10	0	7	0	0	0	0	3	3	10	3	10	0	7	0	0	0	0	3	10	3	3
Campania	2021	15	0	7	0	0	0	0	8	8	15	8	14	0	7	0	0	0	0	7	14	7	7
Puglia	2021	27	0	22	0	0	0	2	3	3	27	5	26	0	21	0	0	0	2	3	24	5	5
Basilicata	2021	15	0	7	0	0	2	0	6	8	13	8	12	0	5	0	0	2	0	5	12	7	5
Calabria	2021	15	0	7	0	0	0	0	8	8	15	8	10	0	5	0	0	0	0	5	10	5	5
Sicily (Sicilia)	2021	13	7	3	0	3	0	0	0	10	6	0	12	6	3	0	3	0	0	0	3	0	3

Sardinia (Sardegna)	2021	29	0	28	0	1	0	0	0	1	29	0	21	0	21	0	0	0	0	0	21	0	0
**All Italy**	**2021**	**567**	**8**	**434**	**0**	**5**	**5**	**14**	**101**	**119**	**554**	**120**	**406**	**7**	**305**	**0**	**4**	**5**	**8**	**77**	**387**	**90**	**89**
Piedmont (Piemonte)	2022	70	3	45	0	15	0	7	0	18	67	7	62	2	41	0	13	0	6	0	15	60	6
Valle d'Aosta	2022	14	0	12	0	2	0	0	0	2	14	0	7	0	6	0	1	0	0	0	1	7	0
Liguria	2022	14	1	10	0	0	0	3	0	1	13	3	8	1	6	0	0	0	1	0	1	7	1
Lombardy (Lombardia)	2022	69	2	56	0	6	0	4	1	9	67	5	55	2	43	0	5	0	4	1	8	53	5
Trentino-Alto Adige	2022	30	0	29	0	0	0	1	0	0	30	1	25	0	24	0	0	0	1	0	0	25	1
Veneto	2022	43	1	32	0	1	0	9	0	2	42	9	38	1	30	0	1	0	6	0	2	37	6
Friuli-Venezia Giulia	2022	17	1	12	0	3	0	1	0	4	16	1	9	1	6	0	1	0	1	0	2	8	1
Emilia-Romagna	2022	263	0	258	0	3	0	2	0	3	263	2	130	0	128	0	1	0	1	0	1	130	1
Tuscany (Toscana)	2022	40	1	30	0	2	0	7	0	3	39	7	35	1	26	0	2	0	6	0	3	34	6
Umbria	2022	5	0	4	0	0	0	0	1	1	5	1	4	0	3	0	0	0	0	1	1	4	1
Marche	2022	10	0	8	0	0	0	0	2	2	10	2	10	0	8	0	0	0	0	2	2	10	2
Lazio	2022	21	0	10	0	0	1	0	10	11	20	11	19	0	10	0	0	1	0	8	9	18	9
Abruzzo	2022	19	0	9	0	0	0	0	10	10	19	10	17	0	9	0	0	0	0	8	8	17	8
Molise	2022	12	0	6	0	0	1	0	5	6	11	6	11	0	5	0	0	1	0	5	6	10	6
Campania	2022	19	0	13	0	0	0	0	6	6	19	6	17	0	12	0	0	0	0	5	5	17	5
Puglia	2022	28	0	13	0	0	0	0	15	15	28	15	23	0	10	0	0	0	0	13	13	23	13
Basilicata	2022	18	0	9	0	0	1	0	8	9	17	9	17	0	8	0	0	1	0	8	9	16	9
Calabria	2022	22	0	10	0	0	2	0	10	12	20	12	21	0	10	0	0	2	0	9	11	19	11
Sicily (Sicilia)	2022	47	29	11	0	7	0	0	0	36	18	0	36	23	7	0	6	0	0	0	29	13	0
Sardinia (Sardegna)	2022	21	0	11	0	0	0	0	10	10	21	10	20	0	10	0	0	0	0	10	10	20	10
**All Italy**	**2022**	**782**	**38**	**588**	**0**	**39**	**5**	**34**	**78**	**160**	**739**	**117**	**564**	**31**	**402**	**0**	**30**	**5**	**26**	**70**	**136**	**528**	**101**
Piedmont (Piemonte)	2023	77	0	53	0	0	0	2	22	22	77	24	24	0	39	0	0	0	2	15	15	56	17
Valle d'Aosta	2023	11	0	10	0	1	0	0	0	1	11	0	0	0	8	0	1	0	0	0	1	9	0
Liguria	2023	14	0	13	0	0	0	0	1	1	14	1	1	0	10	0	0	0	0	1	1	11	1
Lombardy (Lombardia)	2023	67	0	48	0	0	0	3	16	16	67	19	19	0	36	0	0	0	2	14	14	52	16
Trentino-Alto Adige	2023	33	0	33	0	0	0	0	0	0	33	0	0	0	26	0	0	0	0	0	0	26	0
Veneto	2023	36	0	33	1	0	0	0	2	2	35	3	3	0	24	1	0	0	0	1	1	25	2
Friuli-Venezia Giulia	2023	20	0	17	0	0	1	1	1	2	19	3	3	0	14	0	0	1	1	0	1	15	2
Emilia-Romagna	2023	132	0	114	0	3	0	5	10	13	132	15	15	0	65	0	1	0	3	5	6	74	8
Tuscany (Toscana)	2023	37	0	29	0	0	0	4	4	4	37	8	8	0	15	0	0	0	3	3	3	21	6
Umbria	2023	20	0	14	0	0	0	4	2	2	20	6	6	0	8	0	0	0	2	1	1	11	3
Marche	2023	42	0	39	0	1	0	1	1	2	42	2	2	0	18	0	1	0	1	1	2	21	2
Lazio	2023	36	0	22	1	0	0	3	10	10	35	14	14	0	11	1	0	0	3	7	7	21	11
Abruzzo	2023	70	0	42	2	2	0	7	17	19	68	26	26	0	22	1	2	0	4	10	12	38	15
Molise	2023	31	0	19	0	1	0	5	6	7	31	11	11	0	11	0	0	0	3	4	4	18	7
Campania	2023	38	0	26	0	1	0	0	11	12	38	11	11	0	16	0	1	0	0	7	8	24	7
Puglia	2023	84	0	44	0	0	0	6	34	34	84	40	40	0	24	0	0	0	2	15	15	41	17
Basilicata	2023	41	0	21	0	0	0	4	16	16	41	20	20	0	11	0	0	0	4	10	10	25	14
Calabria	2023	16	0	11	0	1	0	3	1	2	16	4	4	0	6	0	1	0	2	1	2	10	3
Sicily (Sicilia)	2023	32	7	6	0	0	1	5	13	21	24	19	19	5	4	0	0	1	5	11	17	20	17
Sardinia (Sardegna)	2023	93	1	81	0	0	0	6	5	6	92	11	11	0	38	0	0	0	2	2	2	42	4
**All Italy**	**2023**	**930**	**8**	**675**	**4**	**10**	**2**	**59**	**172**	**192**	**916**	**237**	**237**	**5**	**406**	**3**	**7**	**2**	**39**	**108**	**122**	**560**	**152**

[1] The 20 Italian regions are listed by the following geographic zones: North (Piedmont, Valle d'Aosta, Liguria, Lombardy, Trentino-Alto Adige, Veneto, Friuli-Venezia Giulia, Emilia-Romagna), Central (Tuscany, Marche, Umbria, Lazio, Abruzzo), South (Molise, Campania, Puglia, Basilicata, Calabria) and the two islands (Sicily and Sardinia). The Italian name of some regions is provided in parentheses.

[2] Years of production.

[3] Total number of samples analysed.

[4] The number of unique honey samples within the same year of production: this number refers to different beekeepers who provided samples in the year of production considered.

Supplementary Table 2. Results of the logistic regression analyses testing the effect of latitude on the distribution of honey samples including some lineages or lineage combinations (various mtDNA lineage profiles or patterns) in the 2018-2023 period or in single years.

Lineages[1]	Years	Macro-regions	Latitude [2]	Chi-Square [3]
Including A	2018-2023	Peninsula + Sicily	-0.280 (0.017); <0.0001	287.81; <0.0001
		Peninsula	-0.222 (0.021); <0.0001	112.45; <0.0001
	2018	Peninsula + Sicily	-0.103 (0.044); 0.0192	5.41; 0.0201
		Peninsula	-0.027 (0.050); 0.5909	0.29; 0.5927
	2019	Peninsula + Sicily	-0.361 (0.049); <0.0001	59.56; <0.0001
		Peninsula	-0.273 (0.069); 0.0001	15.72; 0.0001
	2020	Peninsula + Sicily	-0.173 (0.043); 0.0001	15.16; 0.0001
		Peninsula	-0.032 (0.067); 0.639	0.22; 0.642
	2021	Peninsula + Sicily	-0.306 (0.047); <0.0001	43.99; <0.0001
		Peninsula	-0.279 (0.053); <0.0001	27.70; <0.0001
	2022	Peninsula + Sicily	-0.469 (0.041); <0.0001	161.00; <0.0001
		Peninsula	-0.474 (0.053); <0.0001	86.52; <0.0001
	2023	Peninsula + Sicily	-0.262 (0.037); <0.0001	52.31; <0.0001
		Peninsula	-0.228 (0.044); <0.0001	27.74; <0.0001
Including M	2018-2023	Peninsula + Sicily	-0.183 (0.016); <0.0001	130.10; <0.0001
		Peninsula	-0.266 (0.019); <0.0001	188.27; <0.0001
	2018	Peninsula + Sicily	-0.091 (0.039); 0.021	5.32; 0.021
		Peninsula	-0.081 (0.042); 0.053	3.69; 0.055
	2019	Peninsula + Sicily	-0.182 (0.044); <0.0001	17.06; <0.0001
		Peninsula	-0.258 (0.060); <0.0001	18.66; <0.0001
	2020	Peninsula + Sicily	-0.062 (0.044); 0.156	1.93; 0.165
		Peninsula	-0.207 (0.055); 0.0002	13.50; 0.0002
	2021	Peninsula + Sicily	-0.192 (0.046); <0.0001	17.52; <0.0001
		Peninsula	-0.290 (0.052); <0.0001	31.70; <0.0001
	2022	Peninsula + Sicily	-0.248 (0.038); <0.0001	40.09; <0.0001
		Peninsula	-0.548 (0.056); <0.0001	109.33; <0.0001
	2023	Peninsula + Sicily	-0.257 (0.035); <0.0001	56.26; <0.0001
		Peninsula	-0.260 (0.041); <0.0001	41.49; <0.0001
Only C	2018-2023	Peninsula + Sicily	0.277 (0.016); <0.0001	328.92; <0.0001
		Peninsula	0.242 (0.019); <0.0001	165.34; <0.0001
	2018	Peninsula + Sicily	0.123 (0.038); 0.0011	10.70; 0.0011
		Peninsula	0.063 (0.041); 0.121	2.33; 0.123
	2019	Peninsula +Sicily	0.306 (0.043); <0.0001	55.19; <0.0001
		Peninsula	0.274 (0.057); <0.0001	23.54; <0.0001
	2020	Peninsula + Sicily	0.238 (0.039); <0.0001	35.89; <0.0001
		Peninsula	0.247 (0.054); <0.0001	20.51; <0.0001
	2021	Peninsula + Sicily	0.303 (0.046); <0.0001	45.43; <0.0001
		Peninsula	0.283 (0.052); <0.0001	30.65; <0.0001
	2022	Peninsula + Sicily	0.366 (0.037); <0.0001	112.09; <0.0001
		Peninsula	0.343 (0.047); <0.0001	53.05; <0.0001
	2023	Peninsula + Sicily	0.302 (0.036); <0.0001	78.68; <0.0001
		Peninsula	0.266 (0.041); <0.0001	44.50; <0.0001

[1] Dependent variables were based on honey samples with the following mtDNA lineages or lineage patterns: all honey samples including the A lineage, all honey samples including the M lineage, only C lineage in the honey. The used lineage information was from honey samples produced in all regions of the Italian Peninsula and Sicily. [2] X_1 variable: values indicate regression coefficients, their standard errors (in brackets) and the *P*-values. [3] Values of the Chi-square and the probability of the test in the model.

Supplementary Table S3. Results of the logistic regression analyses testing the effect of latitude, including Sardinia, on the distribution of honey samples including some mtDNA lineages or lineage combinations (various mtDNA lineage profiles or patterns) in the 2018-2023 period or in single years.

Lineages[1]	Years	Macro-regions	Latitude[2]	Chi-Square[3]
A	2018-2023	Peninsula + Sardinia + Sicily	-0.246 (0.016); <0.0001	242.97; <0.0001
		Peninsula + Sardinia	-0.178 (0.019); <0.0001	84.80; <0.0001
	2018	Peninsula + Sardinia + Sicily	-0.101 (0.042); 0.0168	5.61; 0.0178
		Peninsula + Sardinia	-0.027 (0.048); 0.5785	0.31; 0.5805
	2019	Peninsula + Sardinia + Sicily	-0.350 (0.048); <0.0001	59.82; <0.0001
		Peninsula + Sardinia	-0.251 (0.064); 0.0001	15.59; 0.0001
	2020	Peninsula + Sardinia + Sicily	-0.148 (0.042); 0.0004	11.60; 0.0007
		Peninsula + Sardinia	0.002 (0.063); 0.9783	0.01; 0.9783
	2021	Peninsula + Sardinia + Sicily	-0.229 (0.043); <0.0001	28.20; <0.0001
		Peninsula + Sardinia	-0.185 (0.048); 0.0001	14.61; 0.0001
	2022	Peninsula + Sardinia + Sicily	-0.466 (0.034); <0.0001	169.56; <0.0001
		Peninsula + Sardinia	-0.467 (0.049); <0.0001	98.12; <0.0001
	2023	Peninsula + Sardinia + Sicily	-0.018 (0.034); <0.0001	28.07; <0.0001
		Peninsula + Sardinia	-0.120 (0.039); 0.0020	9.66; 0.0019
M	2018-2023	Peninsula + Sardinia + Sicily	-0.156 (0.015); <0.0001	102.27; <0.0001
		Peninsula + Sardinia	-0.209 (0.018); <0.0001	136.64; <0.0001
	2018	Peninsula + Sardinia + Sicily	-0.088 (0.038); 0.019	5.43; 0.0191
		Peninsula + Sardinia	-0.078 (0.040); 0.052	3.72; 0.054
	2019	Peninsula + Sardinia + Sicily	-0.169 (0.043); 0.0001	15.02; 0.0001
		Peninsula + Sardinia	-0.216 (0.056); 0.0001	14.74; 0.0001
	2020	Peninsula + Sardinia + Sicily	-0.032 (0.044); 0.4595	0.53; 0.4644
		Peninsula + Sardinia	-0136 (0.052); 0.008	6.69; 0.0097
	2021	Peninsula + Sardinia + Sicily	-0.122 (0.043); 0.0045	7.90; 0.0049
		Peninsula + Sardinia	-0180 (0.046); 0.0001	14.76; 0.0001
	2022	Peninsula + Sardinia + Sicily	-0.265 (0.037); <0.0001	50.10; <0.0001
		Peninsula + Sardinia	-0535 (0.052); <0.0001	121.58; <0.0001
	2023	Peninsula + Sardinia + Sicily	-0.177 (0.032); <0.0001	31.45; <0.0001
		Peninsula + Sardinia	-0150 (0.036); <0.0001	17.88; <0.0001
C	2018-2023	Peninsula + Sardinia + Sicily	0.239 (0.015); <0.0001	270.40; <0.0001
		Peninsula + Sardinia	0.191 (0.017); <0.0001	121.74; <0.0001
	2018	Peninsula + Sardinia + Sicily	0.118 (0.036); 0.0011	10.64; 0.0011
		Peninsula + Sardinia	0.060 (0.040); 0.1310	2.25; 0.1334
	2019	Peninsula + Sardinia + Sicily	0.293 (0.042); <0.0001	53.50; <0.0001
		Peninsula + Sardinia	0.255 (0.053); <0.0001	23.60; <0.0001
	2020	Peninsula + Sardinia + Sicily	0.203 (0.038); <0.0001	27.92; <0.0001
		Peninsula + Sardinia	0.179 (0.049); 0.0003	12.55; 0.0004
	2021	Peninsula + Sardinia + Sicily	0.221 (0.042); <0.0001	28.04; <0.0001
		Peninsula + Sardinia	0.184 (0.046); 0.0001	15.59; 0.0001
	2022	Peninsula + Sardinia + Sicily	0.362 (0.035); <0.0001	117.80; <0.0001
		Peninsula + Sardinia	0.341 (0.044); <0.0001	60.89; <0.0001
	2023	Peninsula + Sardinia + Sicily	0.211 (0.032); <0.0001	46.36; <0.0001
		Peninsula + Sardinia	0.155 (0.035); <0.0001	19.82; <0.0001

[1] Dependent variable were based on honey samples with the following mitotype lineages or mitotype patterns: only C lineage in the honey, all honey samples including the A lineage, all honey samples including the M lineage. The used mitotype information was from honey samples produced in all regions of the Italian peninsula and Sicily. [2] X_1 variable: values indicate regression coefficients, their standard errors (in brackets) and the *P*-values. [3] Values of the Chi-square and the probability of the test in the model.

Supplementary Table S4. Results of the logistic regression analyses testing the effect of longitude on the distribution of honey samples in the North of Italy including some mtDNA lineages or lineage combinations (patterns or profiles) in the 2018-2023 period or in single years.

**Lineages** [1]	**Years**	**Longitude** [2]	**Chi-Square** [3]
Including A	2018-2023	-0.324 (0.0.40); <0.0001	68.73: <0.0001
	2018	-0.079 (0.093); 0.396	0.72; 0.396
	2019	-0.280 (0.117);0.017	6.06; 0.014
	2020	-0.302 (0.082);0.0002	13.74;0.0002
	2021	-0.606 (0.108); <0.0001	37.09; <0.0001
	2022	-0.424 (0.113); 0.0002	14.25; 0.0002
	2023	-0331 (0.092); 0.0003	13.47; 0.0002
Including M	2018-2023	-0.202 (0.036); <0.0001	31.47; <0.0001
	2018	0.046 (0.077); 0.552	0.35; 0.552
	2019	-0.075 (0.094); 0.421	0.65; 0.421
	2020	-0.265 (0.076); 0.0005	12.16; 0.0005
	2021	-0.533 (0.101); <0.0001	31.55; <0.0001
	2022	-0.183 (0.131); 0.162	1.92; 0.166
	2023	-0.307 (0.087); 0.0004	13.00, 0.0003
Only C	2018-2023	0.216 (0.341); <0.0001	40.778 <0.0001
	2018	-0.069 (0.075); 0.356	0.85; 0.355
	2019	0.130 (0.089); 0.146	2.13; 0.144
	2020	0.265 (0.076); 0.0005	12.16; 0.0005
	2021	0.510 (0.099); <0.0001	29.58; <0.0001
	2022	0.348 (0.090); 0.0001	14.95; 0.0001
	2023	0.286 (0.085); 0.0007	11.86; 0.0006

[1] Dependent variable were based on honey samples with the following mtDNA lineages or lineage patterns: all honey samples including the A lineage, all honey samples including the M lineage or only the C lineage in the honey. [2] X_1 variable: values indicate regression coefficients, their standard errors (in brackets) and the *P*-values. [3] Values of the Chi-square and the probability of the test in the model.

Supplementary Table S5. P-values of the logistic regression conducted over years for the various mtDNA lineage profiles (honey samples containing the A lineage; honey samples containing the M lineage; honey samples with only the C lineage), considering macro-regions.

Macro-regions	A	M	Only C
North	0.788	0.00022	0.013
Central + South	0.000002	0.00008	0.00079

Supplementary Table S6. List of beekeepers who provided honey samples across years (2018-2023).

Region	Beekeeper ID	No. of honey samples submitted	No. of years of submission of honey samples
Abruzzo	35	2	1
Abruzzo	62	3	2
Abruzzo	66	1	1
Abruzzo	87	2	1
Abruzzo	90	1	1
Abruzzo	108	5	4
Abruzzo	113	4	2
Abruzzo	146	5	4
Abruzzo	171	3	2
Abruzzo	179	5	3
Abruzzo	182	1	1
Abruzzo	193	1	1
Abruzzo	203	1	1
Abruzzo	207	6	4
Abruzzo	218	2	1
Abruzzo	219	2	2
Abruzzo	223	2	1
Abruzzo	241	1	1
Abruzzo	264	1	1
Abruzzo	271	2	1
Abruzzo	278	1	1
Abruzzo	294	8	5
Abruzzo	304	1	1
Abruzzo	307	5	4
Abruzzo	317	2	2
Abruzzo	321	1	1
Abruzzo	348	1	1
Abruzzo	357	2	2
Abruzzo	410	1	1
Abruzzo	434	7	5
Abruzzo	438	2	2
Abruzzo	451	4	3
Abruzzo	470	1	1
Abruzzo	485	2	2
Abruzzo	542	3	1
Abruzzo	546	1	1
Abruzzo	583	3	2
Abruzzo	612	1	1
Abruzzo	626	3	3
Abruzzo	628	1	1
Abruzzo	629	1	1
Abruzzo	675	1	1
Abruzzo	677	5	4
Abruzzo	688	1	1

Abruzzo	711	1	1
Abruzzo	773	1	1
Abruzzo	796	2	1
Abruzzo	815	3	2
Abruzzo	816	2	1
Abruzzo	836	3	3
Abruzzo	838	1	1
Abruzzo	865	1	1
Abruzzo	873	3	2
Abruzzo	882	2	1
Abruzzo	913	2	1
Abruzzo	920	1	1
Abruzzo	954	1	1
Abruzzo	984	1	1
Abruzzo	1003	4	1
Abruzzo	1006	1	1
Abruzzo	1008	3	2
Abruzzo	1010	1	1
Abruzzo	1054	1	1
Abruzzo	1113	3	2
Abruzzo	1129	1	1
Abruzzo	1176	2	1
Abruzzo	1186	2	1
Abruzzo	1219	1	1
Abruzzo	1221	1	1
Abruzzo	1239	1	1
Abruzzo	1240	1	1
Abruzzo	1242	1	1
Abruzzo	1243	1	1
Abruzzo	1245	1	1
Basilicata	7	4	3
Basilicata	31	2	2
Basilicata	49	4	4
Basilicata	62	2	2
Basilicata	70	4	4
Basilicata	81	1	1
Basilicata	94	2	2
Basilicata	98	3	3
Basilicata	112	1	1
Basilicata	127	1	1
Basilicata	171	1	1
Basilicata	173	2	1
Basilicata	179	2	2
Basilicata	184	3	2
Basilicata	201	2	2
Basilicata	214	1	1
Basilicata	247	2	2
Basilicata	261	10	4
Basilicata	262	1	1
Basilicata	285	1	1

Basilicata	307	2	2
Basilicata	313	7	4
Basilicata	326	2	2
Basilicata	337	1	1
Basilicata	391	2	2
Basilicata	392	3	2
Basilicata	430	1	1
Basilicata	463	1	1
Basilicata	472	1	1
Basilicata	542	1	1
Basilicata	549	1	1
Basilicata	588	2	1
Basilicata	598	1	1
Basilicata	640	4	3
Basilicata	644	4	3
Basilicata	650	2	1
Basilicata	671	1	1
Basilicata	678	1	1
Basilicata	714	1	1
Basilicata	725	3	2
Basilicata	733	2	2
Basilicata	737	5	3
Basilicata	783	3	3
Basilicata	794	1	1
Basilicata	823	1	1
Basilicata	868	4	3
Basilicata	911	1	1
Basilicata	917	1	1
Basilicata	972	5	3
Basilicata	1057	1	1
Basilicata	1082	3	2
Basilicata	1097	1	1
Basilicata	1106	1	1
Basilicata	1189	1	1
Basilicata	1205	1	1
Basilicata	1220	1	1
Basilicata	1248	1	1
Calabria	17	6	5
Calabria	55	4	3
Calabria	146	1	1
Calabria	152	3	3
Calabria	212	2	2
Calabria	225	5	5
Calabria	254	2	2
Calabria	265	1	1
Calabria	293	1	1
Calabria	346	1	1
Calabria	486	1	1
Calabria	545	2	2
Calabria	551	1	1

Calabria	556	1	1
Calabria	572	1	1
Calabria	573	1	1
Calabria	577	5	4
Calabria	685	2	2
Calabria	699	7	4
Calabria	726	1	1
Calabria	727	4	3
Calabria	729	2	2
Calabria	743	1	1
Calabria	757	1	1
Calabria	760	1	1
Calabria	781	2	2
Calabria	785	1	1
Calabria	801	2	2
Calabria	834	2	2
Calabria	890	1	1
Calabria	894	1	1
Calabria	896	2	2
Calabria	898	1	1
Calabria	902	4	2
Calabria	905	1	1
Calabria	947	2	2
Calabria	1022	2	1
Calabria	1024	1	1
Calabria	1036	1	1
Calabria	1043	1	1
Calabria	1062	1	1
Calabria	1079	1	1
Calabria	1096	4	2
Calabria	1131	1	1
Calabria	1137	1	1
Calabria	1168	1	1
Campania	3	1	1
Campania	8	3	3
Campania	21	4	4
Campania	33	2	2
Campania	101	1	1
Campania	114	4	4
Campania	126	5	4
Campania	194	1	1
Campania	195	1	1
Campania	198	2	2
Campania	201	1	1
Campania	283	1	1
Campania	317	1	1
Campania	328	6	4
Campania	379	1	1
Campania	396	6	5
Campania	407	1	1

Campania	417	1	1
Campania	424	1	1
Campania	427	5	4
Campania	437	1	1
Campania	442	1	1
Campania	498	2	2
Campania	502	6	4
Campania	533	1	1
Campania	535	1	1
Campania	548	2	2
Campania	564	4	3
Campania	587	1	1
Campania	600	1	1
Campania	605	2	2
Campania	606	1	1
Campania	607	2	2
Campania	613	1	1
Campania	619	1	1
Campania	626	1	1
Campania	661	2	2
Campania	666	4	3
Campania	701	1	1
Campania	730	1	1
Campania	745	1	1
Campania	747	2	2
Campania	749	2	1
Campania	752	5	4
Campania	815	1	1
Campania	816	2	1
Campania	829	1	1
Campania	847	2	2
Campania	859	2	2
Campania	864	2	2
Campania	883	3	2
Campania	899	1	1
Campania	952	2	2
Campania	960	1	1
Campania	991	6	2
Campania	1003	1	1
Campania	1020	3	2
Campania	1032	3	2
Campania	1064	1	1
Campania	1094	1	1
Campania	1151	1	1
Campania	1188	1	1
Campania	1202	1	1
Campania	1212	1	1
Emilia-Romagna	2	1	1
Emilia-Romagna	5	4	3
Emilia-Romagna	12	2	1

Emilia-Romagna	24	10	3
Emilia-Romagna	26	4	3
Emilia-Romagna	30	6	6
Emilia-Romagna	41	22	6
Emilia-Romagna	43	9	4
Emilia-Romagna	46	14	5
Emilia-Romagna	49	25	6
Emilia-Romagna	50	8	5
Emilia-Romagna	61	1	1
Emilia-Romagna	76	13	5
Emilia-Romagna	116	1	1
Emilia-Romagna	129	5	5
Emilia-Romagna	137	1	1
Emilia-Romagna	139	3	2
Emilia-Romagna	141	1	1
Emilia-Romagna	147	4	4
Emilia-Romagna	157	10	6
Emilia-Romagna	158	4	1
Emilia-Romagna	159	15	6
Emilia-Romagna	180	1	1
Emilia-Romagna	181	4	3
Emilia-Romagna	183	9	5
Emilia-Romagna	184	5	4
Emilia-Romagna	186	4	3
Emilia-Romagna	188	2	1
Emilia-Romagna	194	15	6
Emilia-Romagna	199	16	6
Emilia-Romagna	202	3	2
Emilia-Romagna	204	3	3
Emilia-Romagna	208	9	3
Emilia-Romagna	213	4	1
Emilia-Romagna	229	8	4
Emilia-Romagna	230	4	4
Emilia-Romagna	232	1	1
Emilia-Romagna	238	2	1
Emilia-Romagna	239	4	2
Emilia-Romagna	240	5	3
Emilia-Romagna	256	2	1
Emilia-Romagna	260	10	6
Emilia-Romagna	273	1	1
Emilia-Romagna	282	2	2
Emilia-Romagna	287	6	3
Emilia-Romagna	289	1	1
Emilia-Romagna	298	2	1
Emilia-Romagna	315	5	3
Emilia-Romagna	320	1	1
Emilia-Romagna	327	1	1
Emilia-Romagna	329	4	2
Emilia-Romagna	340	6	3
Emilia-Romagna	355	1	1

Emilia-Romagna	366	5	2
Emilia-Romagna	394	1	1
Emilia-Romagna	412	2	2
Emilia-Romagna	413	9	6
Emilia-Romagna	419	2	2
Emilia-Romagna	446	4	2
Emilia-Romagna	460	1	1
Emilia-Romagna	468	1	1
Emilia-Romagna	481	1	1
Emilia-Romagna	490	1	1
Emilia-Romagna	492	7	4
Emilia-Romagna	503	2	1
Emilia-Romagna	505	1	1
Emilia-Romagna	506	2	1
Emilia-Romagna	526	24	4
Emilia-Romagna	540	1	1
Emilia-Romagna	543	4	2
Emilia-Romagna	544	2	1
Emilia-Romagna	569	7	3
Emilia-Romagna	580	4	3
Emilia-Romagna	590	1	1
Emilia-Romagna	603	8	4
Emilia-Romagna	604	1	1
Emilia-Romagna	621	1	1
Emilia-Romagna	624	1	1
Emilia-Romagna	631	1	1
Emilia-Romagna	638	1	1
Emilia-Romagna	655	2	2
Emilia-Romagna	657	1	1
Emilia-Romagna	673	1	1
Emilia-Romagna	686	1	1
Emilia-Romagna	691	1	1
Emilia-Romagna	712	1	1
Emilia-Romagna	734	3	3
Emilia-Romagna	764	1	1
Emilia-Romagna	766	2	1
Emilia-Romagna	778	1	1
Emilia-Romagna	805	1	1
Emilia-Romagna	825	1	1
Emilia-Romagna	828	2	1
Emilia-Romagna	840	1	1
Emilia-Romagna	851	5	2
Emilia-Romagna	852	3	1
Emilia-Romagna	855	5	3
Emilia-Romagna	862	3	2
Emilia-Romagna	874	1	1
Emilia-Romagna	884	2	1
Emilia-Romagna	889	2	1
Emilia-Romagna	924	1	1
Emilia-Romagna	942	1	1

Emilia-Romagna	946	2	1
Emilia-Romagna	985	8	3
Emilia-Romagna	1001	4	1
Emilia-Romagna	1018	2	1
Emilia-Romagna	1025	2	1
Emilia-Romagna	1026	1	1
Emilia-Romagna	1031	3	2
Emilia-Romagna	1033	1	1
Emilia-Romagna	1035	1	1
Emilia-Romagna	1040	1	1
Emilia-Romagna	1045	1	1
Emilia-Romagna	1055	1	1
Emilia-Romagna	1089	1	1
Emilia-Romagna	1105	1	1
Emilia-Romagna	1114	2	1
Emilia-Romagna	1115	2	1
Emilia-Romagna	1172	1	1
Emilia-Romagna	1197	1	1
Emilia-Romagna	1204	1	1
Emilia-Romagna	1250	1	1
Emilia-Romagna	1251	3	3
Emilia-Romagna	1252	8	4
Emilia-Romagna	1253	3	2
Emilia-Romagna	1254	1	1
Emilia-Romagna	1255	4	1
Emilia-Romagna	1256	12	4
Emilia-Romagna	1257	1	1
Emilia-Romagna	1258	3	1
Emilia-Romagna	1259	2	2
Emilia-Romagna	1260	2	2
Emilia-Romagna	1261	2	1
Emilia-Romagna	1262	1	1
Emilia-Romagna	1263	2	2
Emilia-Romagna	1264	2	2
Emilia-Romagna	1265	2	1
Emilia-Romagna	1266	13	4
Emilia-Romagna	1267	4	3
Emilia-Romagna	1268	8	3
Emilia-Romagna	1269	6	4
Emilia-Romagna	1270	1	1
Emilia-Romagna	1271	3	2
Emilia-Romagna	1272	2	1
Emilia-Romagna	1273	1	1
Emilia-Romagna	1274	11	3
Emilia-Romagna	1275	2	1
Emilia-Romagna	1276	1	1
Emilia-Romagna	1277	1	1
Emilia-Romagna	1278	1	1
Emilia-Romagna	1279	1	1
Emilia-Romagna	1280	1	1

Emilia-Romagna	1281	1	1
Emilia-Romagna	1282	6	4
Emilia-Romagna	1283	2	2
Emilia-Romagna	1284	1	1
Emilia-Romagna	1285	1	1
Emilia-Romagna	1286	7	3
Emilia-Romagna	1287	2	1
Emilia-Romagna	1288	3	2
Emilia-Romagna	1289	7	2
Emilia-Romagna	1290	1	1
Emilia-Romagna	1291	3	2
Emilia-Romagna	1292	2	1
Emilia-Romagna	1293	1	1
Emilia-Romagna	1294	1	1
Emilia-Romagna	1295	1	1
Emilia-Romagna	1296	1	1
Emilia-Romagna	1297	2	2
Emilia-Romagna	1298	6	3
Emilia-Romagna	1299	4	3
Emilia-Romagna	1300	1	1
Emilia-Romagna	1301	1	1
Emilia-Romagna	1302	1	1
Emilia-Romagna	1303	7	4
Emilia-Romagna	1304	2	2
Emilia-Romagna	1305	2	1
Emilia-Romagna	1306	2	1
Emilia-Romagna	1307	2	2
Emilia-Romagna	1308	16	3
Emilia-Romagna	1309	3	3
Emilia-Romagna	1310	1	1
Emilia-Romagna	1311	1	1
Emilia-Romagna	1312	5	1
Emilia-Romagna	1313	2	1
Emilia-Romagna	1314	2	1
Emilia-Romagna	1315	1	1
Emilia-Romagna	1316	1	1
Emilia-Romagna	1317	1	1
Emilia-Romagna	1318	6	4
Emilia-Romagna	1319	3	1
Emilia-Romagna	1320	3	1
Emilia-Romagna	1321	1	1
Emilia-Romagna	1322	3	1
Emilia-Romagna	1323	4	3
Emilia-Romagna	1324	1	1
Emilia-Romagna	1325	7	3
Emilia-Romagna	1326	1	1
Emilia-Romagna	1327	1	1
Emilia-Romagna	1328	2	2
Emilia-Romagna	1329	1	1
Emilia-Romagna	1330	3	1

Emilia-Romagna	1331	4	3
Emilia-Romagna	1332	1	1
Emilia-Romagna	1333	5	4
Emilia-Romagna	1334	2	1
Emilia-Romagna	1335	4	3
Emilia-Romagna	1336	3	2
Emilia-Romagna	1337	1	1
Emilia-Romagna	1338	2	2
Emilia-Romagna	1339	1	1
Emilia-Romagna	1340	2	1
Emilia-Romagna	1341	1	1
Emilia-Romagna	1342	1	1
Emilia-Romagna	1343	2	1
Emilia-Romagna	1344	3	1
Emilia-Romagna	1345	15	4
Emilia-Romagna	1346	3	3
Emilia-Romagna	1347	4	2
Emilia-Romagna	1348	1	1
Emilia-Romagna	1349	1	1
Emilia-Romagna	1350	4	2
Emilia-Romagna	1351	1	1
Emilia-Romagna	1352	2	1
Emilia-Romagna	1353	4	2
Emilia-Romagna	1354	2	2
Emilia-Romagna	1355	4	2
Emilia-Romagna	1356	3	1
Emilia-Romagna	1357	1	1
Emilia-Romagna	1358	10	3
Emilia-Romagna	1359	1	1
Emilia-Romagna	1360	3	1
Emilia-Romagna	1361	4	2
Emilia-Romagna	1362	8	3
Emilia-Romagna	1363	1	1
Emilia-Romagna	1364	1	1
Emilia-Romagna	1365	2	1
Emilia-Romagna	1366	1	1
Emilia-Romagna	1367	9	2
Emilia-Romagna	1368	4	2
Emilia-Romagna	1369	2	2
Emilia-Romagna	1370	3	1
Emilia-Romagna	1371	4	2
Emilia-Romagna	1372	1	1
Emilia-Romagna	1373	14	3
Emilia-Romagna	1374	2	1
Emilia-Romagna	1375	1	1
Emilia-Romagna	1376	1	1
Emilia-Romagna	1378	2	1
Emilia-Romagna	1379	1	1
Friuli-Venezia Giulia	86	14	5
Friuli-Venezia Giulia	135	1	1

Friuli-Venezia Giulia	150	1	1
Friuli-Venezia Giulia	167	3	3
Friuli-Venezia Giulia	175	4	2
Friuli-Venezia Giulia	200	8	4
Friuli-Venezia Giulia	210	4	3
Friuli-Venezia Giulia	236	2	2
Friuli-Venezia Giulia	250	18	5
Friuli-Venezia Giulia	278	8	4
Friuli-Venezia Giulia	302	2	2
Friuli-Venezia Giulia	331	1	1
Friuli-Venezia Giulia	333	1	1
Friuli-Venezia Giulia	349	1	1
Friuli-Venezia Giulia	350	1	1
Friuli-Venezia Giulia	389	1	1
Friuli-Venezia Giulia	403	1	1
Friuli-Venezia Giulia	414	1	1
Friuli-Venezia Giulia	418	1	1
Friuli-Venezia Giulia	455	5	3
Friuli-Venezia Giulia	471	1	1
Friuli-Venezia Giulia	475	6	3
Friuli-Venezia Giulia	494	1	1
Friuli-Venezia Giulia	504	16	4
Friuli-Venezia Giulia	558	1	1
Friuli-Venezia Giulia	648	2	2
Friuli-Venezia Giulia	665	1	1
Friuli-Venezia Giulia	705	4	3
Friuli-Venezia Giulia	765	2	2
Friuli-Venezia Giulia	806	2	1
Friuli-Venezia Giulia	826	3	2
Friuli-Venezia Giulia	843	2	2
Friuli-Venezia Giulia	922	5	3
Friuli-Venezia Giulia	925	2	1
Friuli-Venezia Giulia	986	4	2
Friuli-Venezia Giulia	1039	4	2
Friuli-Venezia Giulia	1108	2	1
Friuli-Venezia Giulia	1146	1	1
Friuli-Venezia Giulia	1187	1	1
Friuli-Venezia Giulia	1230	2	1
Friuli-Venezia Giulia	1232	1	1
Friuli-Venezia Giulia	1235	1	1
Friuli-Venezia Giulia	1242	1	1
Lazio	14	2	2
Lazio	19	1	1
Lazio	58	2	2
Lazio	62	8	6
Lazio	65	1	1
Lazio	68	2	2
Lazio	74	1	1
Lazio	77	6	4
Lazio	91	6	4

Lazio	114	12	6
Lazio	128	4	1
Lazio	161	1	1
Lazio	171	6	6
Lazio	174	4	3
Lazio	179	1	1
Lazio	237	1	1
Lazio	246	2	2
Lazio	253	4	3
Lazio	263	2	2
Lazio	272	1	1
Lazio	286	3	2
Lazio	307	1	1
Lazio	319	3	3
Lazio	341	1	1
Lazio	342	1	1
Lazio	361	1	1
Lazio	420	1	1
Lazio	422	2	2
Lazio	436	1	1
Lazio	469	4	4
Lazio	517	2	2
Lazio	521	3	3
Lazio	525	1	1
Lazio	530	3	2
Lazio	531	1	1
Lazio	534	1	1
Lazio	561	4	4
Lazio	566	9	5
Lazio	584	1	1
Lazio	591	3	3
Lazio	626	2	2
Lazio	658	1	1
Lazio	683	1	1
Lazio	684	1	1
Lazio	689	1	1
Lazio	716	2	2
Lazio	720	3	2
Lazio	751	1	1
Lazio	754	3	2
Lazio	769	1	1
Lazio	774	3	3
Lazio	793	1	1
Lazio	808	1	1
Lazio	818	1	1
Lazio	866	1	1
Lazio	869	1	1
Lazio	895	1	1
Lazio	908	1	1
Lazio	937	1	1

Lazio	939	2	2
Lazio	950	4	3
Lazio	958	1	1
Lazio	971	1	1
Lazio	973	1	1
Lazio	983	1	1
Lazio	1053	1	1
Lazio	1069	4	2
Lazio	1071	1	1
Lazio	1084	1	1
Lazio	1103	1	1
Lazio	1179	4	1
Lazio	1198	1	1
Lazio	1218	2	1
Lazio	1249	2	1
Lazio	1253	1	1
Lazio	1254	1	1
Lazio	1255	1	1
Liguria	20	3	3
Liguria	54	8	4
Liguria	105	6	4
Liguria	137	3	3
Liguria	215	1	1
Liguria	301	1	1
Liguria	378	1	1
Liguria	465	1	1
Liguria	482	1	1
Liguria	493	1	1
Liguria	536	1	1
Liguria	641	6	4
Liguria	652	1	1
Liguria	667	1	1
Liguria	710	2	2
Liguria	803	7	4
Liguria	807	1	1
Liguria	845	1	1
Liguria	901	3	2
Liguria	910	3	3
Liguria	944	1	1
Liguria	949	1	1
Liguria	964	1	1
Liguria	985	1	1
Liguria	1028	2	1
Liguria	1102	2	2
Liguria	1256	1	1
Liguria	1257	1	1
Liguria	1377	1	1
Lombardy	2	8	5
Lombardy	4	1	1
Lombardy	6	6	6

Lombardy	13	2	2
Lombardy	22	6	4
Lombardy	25	1	1
Lombardy	26	5	4
Lombardy	29	1	1
Lombardy	36	1	1
Lombardy	37	1	1
Lombardy	42	2	2
Lombardy	67	2	2
Lombardy	69	2	2
Lombardy	79	1	1
Lombardy	88	6	3
Lombardy	95	7	6
Lombardy	107	2	2
Lombardy	110	5	4
Lombardy	125	2	2
Lombardy	129	9	6
Lombardy	137	13	6
Lombardy	142	7	6
Lombardy	151	12	6
Lombardy	176	8	6
Lombardy	178	5	3
Lombardy	194	1	1
Lombardy	196	2	2
Lombardy	209	10	6
Lombardy	224	1	1
Lombardy	228	6	5
Lombardy	243	1	1
Lombardy	244	2	2
Lombardy	252	4	3
Lombardy	264	3	2
Lombardy	266	6	6
Lombardy	268	1	1
Lombardy	269	1	1
Lombardy	274	1	1
Lombardy	277	3	3
Lombardy	281	1	1
Lombardy	282	8	4
Lombardy	292	2	2
Lombardy	299	1	1
Lombardy	312	6	5
Lombardy	330	1	1
Lombardy	332	4	4
Lombardy	334	1	1
Lombardy	335	7	6
Lombardy	339	1	1
Lombardy	344	1	1
Lombardy	345	1	1
Lombardy	351	4	3
Lombardy	358	11	6

Lombardy	359	1	1
Lombardy	363	2	2
Lombardy	366	1	1
Lombardy	367	3	2
Lombardy	368	1	1
Lombardy	371	1	1
Lombardy	390	4	4
Lombardy	404	1	1
Lombardy	408	2	2
Lombardy	411	1	1
Lombardy	423	1	1
Lombardy	425	1	1
Lombardy	429	1	1
Lombardy	432	1	1
Lombardy	433	5	3
Lombardy	439	1	1
Lombardy	454	3	2
Lombardy	467	1	1
Lombardy	482	8	6
Lombardy	484	1	1
Lombardy	499	4	4
Lombardy	507	2	2
Lombardy	522	1	1
Lombardy	568	1	1
Lombardy	592	5	5
Lombardy	595	2	2
Lombardy	614	2	1
Lombardy	617	1	1
Lombardy	633	1	1
Lombardy	636	1	1
Lombardy	637	1	1
Lombardy	642	4	3
Lombardy	649	4	3
Lombardy	672	2	2
Lombardy	690	2	2
Lombardy	692	2	2
Lombardy	700	1	1
Lombardy	703	1	1
Lombardy	718	2	2
Lombardy	762	1	1
Lombardy	763	1	1
Lombardy	764	2	2
Lombardy	768	1	1
Lombardy	805	4	3
Lombardy	811	2	2
Lombardy	821	5	4
Lombardy	840	4	3
Lombardy	841	1	1
Lombardy	852	6	4
Lombardy	857	7	4

Lombardy	863	1	1
Lombardy	870	1	1
Lombardy	872	1	1
Lombardy	880	1	1
Lombardy	885	3	2
Lombardy	888	6	3
Lombardy	891	3	2
Lombardy	892	1	1
Lombardy	904	2	2
Lombardy	915	2	2
Lombardy	919	2	1
Lombardy	921	1	1
Lombardy	926	3	3
Lombardy	927	1	1
Lombardy	929	1	1
Lombardy	931	2	2
Lombardy	932	1	1
Lombardy	938	1	1
Lombardy	940	3	3
Lombardy	941	1	1
Lombardy	948	1	1
Lombardy	955	1	1
Lombardy	961	3	3
Lombardy	978	1	1
Lombardy	979	2	1
Lombardy	980	2	1
Lombardy	987	1	1
Lombardy	1015	1	1
Lombardy	1029	1	1
Lombardy	1044	2	2
Lombardy	1046	2	1
Lombardy	1048	1	1
Lombardy	1067	2	2
Lombardy	1068	1	1
Lombardy	1078	1	1
Lombardy	1092	1	1
Lombardy	1093	1	1
Lombardy	1107	1	1
Lombardy	1117	1	1
Lombardy	1119	1	1
Lombardy	1121	1	1
Lombardy	1125	2	2
Lombardy	1132	1	1
Lombardy	1144	1	1
Lombardy	1145	1	1
Lombardy	1154	1	1
Lombardy	1158	1	1
Lombardy	1163	1	1
Lombardy	1164	1	1
Lombardy	1178	1	1

Lombardy	1183	1	1
Lombardy	1192	1	1
Lombardy	1204	1	1
Lombardy	1211	1	1
Lombardy	1227	1	1
Lombardy	1238	1	1
Lombardy	1241	1	1
Lombardy	1246	1	1
Marche	45	1	1
Marche	49	1	1
Marche	62	4	3
Marche	92	4	4
Marche	136	2	2
Marche	157	4	4
Marche	166	2	2
Marche	171	2	2
Marche	179	2	2
Marche	181	5	5
Marche	182	1	1
Marche	186	2	2
Marche	194	10	4
Marche	205	1	1
Marche	207	3	3
Marche	230	3	3
Marche	288	2	2
Marche	298	1	1
Marche	306	1	1
Marche	307	2	2
Marche	308	6	4
Marche	382	1	1
Marche	395	1	1
Marche	397	2	2
Marche	416	3	3
Marche	422	2	2
Marche	515	5	4
Marche	526	2	2
Marche	565	1	1
Marche	602	1	1
Marche	609	2	2
Marche	626	3	2
Marche	635	1	1
Marche	677	4	4
Marche	688	3	3
Marche	788	1	1
Marche	796	4	2
Marche	809	6	2
Marche	815	1	1
Marche	816	2	2
Marche	827	3	2
Marche	856	2	2

Marche	862	1	1
Marche	953	3	2
Marche	976	1	1
Marche	1034	1	1
Marche	1052	5	2
Marche	1090	1	1
Marche	1156	1	1
Marche	1247	3	1
Marche	1258	1	1
Marche	1259	1	1
Molise	49	6	5
Molise	62	5	3
Molise	113	4	2
Molise	134	8	6
Molise	146	5	4
Molise	171	3	2
Molise	179	4	4
Molise	193	1	1
Molise	248	5	4
Molise	270	1	1
Molise	307	6	4
Molise	387	1	1
Molise	514	4	3
Molise	594	2	2
Molise	605	2	2
Molise	626	3	3
Molise	721	3	3
Molise	755	2	2
Molise	815	1	1
Molise	844	1	1
Molise	850	1	1
Molise	873	1	1
Molise	882	3	2
Molise	935	2	2
Molise	951	3	3
Molise	952	5	3
Molise	1003	1	1
Molise	1184	2	1
Molise	1251	1	1
Piedmont	11	3	3
Piedmont	27	7	4
Piedmont	28	6	5
Piedmont	44	1	1
Piedmont	49	2	2
Piedmont	51	2	2
Piedmont	59	4	3
Piedmont	63	4	3
Piedmont	75	4	4
Piedmont	78	11	6
Piedmont	84	2	2

Piedmont	85	1	1
Piedmont	96	2	2
Piedmont	102	2	2
Piedmont	103	1	1
Piedmont	140	2	2
Piedmont	143	5	5
Piedmont	144	2	2
Piedmont	146	1	1
Piedmont	156	9	6
Piedmont	184	1	1
Piedmont	185	3	3
Piedmont	206	3	2
Piedmont	208	3	3
Piedmont	215	2	2
Piedmont	216	3	2
Piedmont	217	1	1
Piedmont	234	3	3
Piedmont	245	6	4
Piedmont	249	4	3
Piedmont	252	13	6
Piedmont	268	1	1
Piedmont	271	2	2
Piedmont	275	2	2
Piedmont	279	10	6
Piedmont	280	7	6
Piedmont	284	2	2
Piedmont	291	8	6
Piedmont	297	4	3
Piedmont	312	2	2
Piedmont	322	3	3
Piedmont	336	1	1
Piedmont	338	5	5
Piedmont	354	1	1
Piedmont	377	1	1
Piedmont	380	1	1
Piedmont	381	1	1
Piedmont	390	1	1
Piedmont	400	1	1
Piedmont	401	3	3
Piedmont	402	1	1
Piedmont	405	1	1
Piedmont	406	1	1
Piedmont	409	1	1
Piedmont	422	1	1
Piedmont	440	6	5
Piedmont	441	5	5
Piedmont	449	1	1
Piedmont	456	2	2
Piedmont	457	1	1
Piedmont	474	1	1

Piedmont	477	3	3
Piedmont	489	1	1
Piedmont	493	1	1
Piedmont	495	4	4
Piedmont	496	1	1
Piedmont	509	2	2
Piedmont	516	1	1
Piedmont	519	3	2
Piedmont	529	2	2
Piedmont	538	1	1
Piedmont	554	1	1
Piedmont	576	2	2
Piedmont	589	3	3
Piedmont	599	2	2
Piedmont	608	6	5
Piedmont	610	1	1
Piedmont	614	3	3
Piedmont	615	2	2
Piedmont	616	1	1
Piedmont	618	1	1
Piedmont	628	1	1
Piedmont	639	1	1
Piedmont	642	1	1
Piedmont	643	1	1
Piedmont	652	2	2
Piedmont	660	2	1
Piedmont	668	1	1
Piedmont	679	2	2
Piedmont	681	2	2
Piedmont	687	1	1
Piedmont	695	1	1
Piedmont	696	2	2
Piedmont	717	4	4
Piedmont	735	2	1
Piedmont	736	3	3
Piedmont	738	4	4
Piedmont	739	6	4
Piedmont	741	3	3
Piedmont	742	1	1
Piedmont	748	2	1
Piedmont	753	1	1
Piedmont	761	1	1
Piedmont	779	1	1
Piedmont	780	1	1
Piedmont	790	1	1
Piedmont	792	3	3
Piedmont	795	1	1
Piedmont	798	1	1
Piedmont	803	1	1
Piedmont	812	7	4

Piedmont	817	10	4
Piedmont	830	4	2
Piedmont	831	1	1
Piedmont	835	1	1
Piedmont	842	1	1
Piedmont	861	2	2
Piedmont	875	1	1
Piedmont	886	3	2
Piedmont	903	1	1
Piedmont	907	4	2
Piedmont	936	1	1
Piedmont	956	1	1
Piedmont	962	3	2
Piedmont	965	2	2
Piedmont	966	1	1
Piedmont	967	6	3
Piedmont	974	3	2
Piedmont	977	2	2
Piedmont	980	1	1
Piedmont	993	1	1
Piedmont	998	1	1
Piedmont	1004	2	2
Piedmont	1005	3	2
Piedmont	1009	1	1
Piedmont	1023	1	1
Piedmont	1027	1	1
Piedmont	1047	2	2
Piedmont	1051	1	1
Piedmont	1058	1	1
Piedmont	1065	3	2
Piedmont	1072	1	1
Piedmont	1087	1	1
Piedmont	1095	3	2
Piedmont	1099	1	1
Piedmont	1100	1	1
Piedmont	1111	1	1
Piedmont	1112	1	1
Piedmont	1126	1	1
Piedmont	1148	2	1
Piedmont	1150	1	1
Piedmont	1152	1	1
Piedmont	1153	2	1
Piedmont	1155	1	1
Piedmont	1160	2	1
Piedmont	1169	1	1
Piedmont	1171	1	1
Piedmont	1177	1	1
Piedmont	1180	1	1
Piedmont	1182	1	1
Piedmont	1196	1	1

Piedmont	1200	1	1
Piedmont	1203	1	1
Piedmont	1217	1	1
Piedmont	1222	1	1
Piedmont	1223	1	1
Piedmont	1224	1	1
Piedmont	1240	1	1
Puglia	7	6	3
Puglia	8	2	2
Puglia	31	5	2
Puglia	62	3	2
Puglia	71	2	2
Puglia	72	9	4
Puglia	97	3	3
Puglia	98	3	3
Puglia	112	6	5
Puglia	117	1	1
Puglia	146	3	3
Puglia	160	1	1
Puglia	171	4	3
Puglia	173	6	5
Puglia	179	2	2
Puglia	197	1	1
Puglia	201	3	3
Puglia	207	3	3
Puglia	214	3	2
Puglia	230	2	2
Puglia	251	1	1
Puglia	255	2	2
Puglia	270	1	1
Puglia	305	2	2
Puglia	307	2	2
Puglia	314	3	3
Puglia	337	1	1
Puglia	383	6	5
Puglia	421	7	6
Puglia	424	1	1
Puglia	476	4	3
Puglia	485	2	2
Puglia	487	2	2
Puglia	539	1	1
Puglia	548	2	2
Puglia	552	1	1
Puglia	555	1	1
Puglia	559	1	1
Puglia	560	1	1
Puglia	562	1	1
Puglia	570	1	1
Puglia	578	5	4
Puglia	582	6	5

Puglia	585	5	3
Puglia	588	4	4
Puglia	626	3	1
Puglia	630	2	2
Puglia	645	2	2
Puglia	646	3	2
Puglia	650	1	1
Puglia	653	5	4
Puglia	678	2	2
Puglia	693	1	1
Puglia	698	3	3
Puglia	706	5	4
Puglia	707	3	2
Puglia	722	2	1
Puglia	749	2	1
Puglia	782	4	3
Puglia	789	3	3
Puglia	794	2	1
Puglia	815	9	1
Puglia	816	11	3
Puglia	829	1	1
Puglia	850	3	2
Puglia	854	2	2
Puglia	860	1	1
Puglia	883	3	3
Puglia	911	2	2
Puglia	912	1	1
Puglia	923	1	1
Puglia	933	1	1
Puglia	994	1	1
Puglia	1013	1	1
Puglia	1016	2	1
Puglia	1139	2	1
Puglia	1157	1	1
Puglia	1161	2	1
Puglia	1170	1	1
Puglia	1219	2	1
Puglia	1244	1	1
Puglia	1260	1	1
Puglia	1261	1	1
Puglia	1262	1	1
Sardinia	1	2	2
Sardinia	10	7	5
Sardinia	15	1	1
Sardinia	38	6	3
Sardinia	39	1	1
Sardinia	89	13	6
Sardinia	93	10	6
Sardinia	99	2	2
Sardinia	104	5	4

Sardinia	130	4	3
Sardinia	131	8	5
Sardinia	133	4	4
Sardinia	148	6	3
Sardinia	154	9	5
Sardinia	155	2	2
Sardinia	162	7	3
Sardinia	189	2	1
Sardinia	231	1	1
Sardinia	242	1	1
Sardinia	257	6	4
Sardinia	300	3	3
Sardinia	316	11	5
Sardinia	393	8	1
Sardinia	444	6	4
Sardinia	461	2	2
Sardinia	462	1	1
Sardinia	488	2	2
Sardinia	527	1	1
Sardinia	541	1	1
Sardinia	547	1	1
Sardinia	611	3	2
Sardinia	647	7	5
Sardinia	669	1	1
Sardinia	708	1	1
Sardinia	724	2	2
Sardinia	746	6	4
Sardinia	767	3	3
Sardinia	775	1	1
Sardinia	776	3	2
Sardinia	786	3	2
Sardinia	791	5	2
Sardinia	833	1	1
Sardinia	839	2	1
Sardinia	877	1	1
Sardinia	878	4	2
Sardinia	881	6	3
Sardinia	893	9	2
Sardinia	897	1	1
Sardinia	900	4	3
Sardinia	906	2	1
Sardinia	909	1	1
Sardinia	943	4	2
Sardinia	963	2	2
Sardinia	996	2	2
Sardinia	1007	1	1
Sardinia	1050	2	1
Sardinia	1061	2	1
Sardinia	1066	3	1
Sardinia	1116	2	2

Sardinia	1118	1	1
Sardinia	1138	2	1
Sardinia	1159	1	1
Sardinia	1162	1	1
Sardinia	1167	1	1
Sardinia	1174	1	1
Sardinia	1175	1	1
Sardinia	1233	1	1
Sardinia	1249	1	1
Sardinia	1250	1	1
Sicily	9	5	4
Sicily	16	1	1
Sicily	34	3	3
Sicily	64	6	5
Sicily	109	7	5
Sicily	111	1	1
Sicily	119	10	6
Sicily	132	3	3
Sicily	145	1	1
Sicily	163	3	2
Sicily	164	1	1
Sicily	177	4	2
Sicily	211	2	2
Sicily	222	1	1
Sicily	259	3	2
Sicily	290	5	4
Sicily	296	8	4
Sicily	318	7	5
Sicily	323	1	1
Sicily	353	1	1
Sicily	356	1	1
Sicily	375	1	1
Sicily	376	1	1
Sicily	398	1	1
Sicily	435	5	5
Sicily	458	2	2
Sicily	459	3	3
Sicily	473	2	2
Sicily	497	2	2
Sicily	500	2	2
Sicily	510	9	5
Sicily	532	1	1
Sicily	553	1	1
Sicily	557	6	5
Sicily	571	1	1
Sicily	574	2	2
Sicily	593	1	1
Sicily	597	2	1
Sicily	627	1	1
Sicily	632	1	1

Sicily	656	1	1
Sicily	663	3	3
Sicily	664	1	1
Sicily	676	1	1
Sicily	694	2	2
Sicily	697	1	1
Sicily	702	1	1
Sicily	704	1	1
Sicily	709	1	1
Sicily	728	1	1
Sicily	732	4	4
Sicily	750	5	4
Sicily	759	5	4
Sicily	819	2	2
Sicily	837	3	2
Sicily	846	3	2
Sicily	867	1	1
Sicily	871	1	1
Sicily	876	2	2
Sicily	957	3	3
Sicily	988	1	1
Sicily	989	1	1
Sicily	992	1	1
Sicily	997	1	1
Sicily	1019	2	1
Sicily	1077	1	1
Sicily	1086	1	1
Sicily	1091	1	1
Sicily	1101	2	2
Sicily	1109	1	1
Sicily	1124	1	1
Sicily	1136	1	1
Sicily	1141	1	1
Sicily	1142	1	1
Sicily	1147	2	1
Sicily	1149	1	1
Sicily	1165	1	1
Sicily	1199	1	1
Sicily	1226	1	1
Sicily	1229	2	1
Sicily	1231	1	1
Sicily	1246	1	1
Sicily	1247	1	1
Sicily	1248	1	1
Tuscany	12	1	1
Tuscany	23	3	3
Tuscany	49	2	2
Tuscany	53	2	2
Tuscany	73	3	3
Tuscany	80	1	1

Tuscany	82	1	1
Tuscany	83	2	2
Tuscany	116	8	6
Tuscany	122	4	3
Tuscany	137	3	3
Tuscany	147	4	4
Tuscany	157	6	6
Tuscany	158	1	1
Tuscany	168	4	3
Tuscany	170	10	6
Tuscany	172	12	6
Tuscany	181	2	2
Tuscany	187	3	3
Tuscany	230	1	1
Tuscany	237	1	1
Tuscany	238	1	1
Tuscany	239	1	1
Tuscany	258	1	1
Tuscany	267	1	1
Tuscany	347	1	1
Tuscany	352	1	1
Tuscany	360	1	1
Tuscany	385	1	1
Tuscany	386	1	1
Tuscany	388	1	1
Tuscany	399	3	3
Tuscany	443	2	2
Tuscany	478	1	1
Tuscany	480	3	3
Tuscany	483	2	2
Tuscany	492	3	3
Tuscany	501	1	1
Tuscany	506	1	1
Tuscany	512	1	1
Tuscany	521	8	4
Tuscany	524	1	1
Tuscany	537	2	2
Tuscany	579	3	3
Tuscany	620	1	1
Tuscany	622	1	1
Tuscany	623	3	3
Tuscany	634	1	1
Tuscany	649	1	1
Tuscany	654	1	1
Tuscany	659	4	4
Tuscany	680	1	1
Tuscany	756	2	2
Tuscany	771	1	1
Tuscany	772	1	1
Tuscany	787	1	1

Tuscany	804	1	1
Tuscany	813	1	1
Tuscany	820	3	3
Tuscany	824	1	1
Tuscany	853	1	1
Tuscany	855	1	1
Tuscany	887	2	2
Tuscany	914	1	1
Tuscany	916	4	3
Tuscany	928	1	1
Tuscany	930	2	2
Tuscany	945	2	1
Tuscany	959	2	2
Tuscany	964	1	1
Tuscany	969	1	1
Tuscany	970	1	1
Tuscany	995	2	2
Tuscany	1000	3	2
Tuscany	1002	2	1
Tuscany	1017	1	1
Tuscany	1037	2	1
Tuscany	1044	1	1
Tuscany	1049	1	1
Tuscany	1059	1	1
Tuscany	1060	1	1
Tuscany	1080	4	2
Tuscany	1085	2	2
Tuscany	1098	1	1
Tuscany	1122	1	1
Tuscany	1127	1	1
Tuscany	1134	2	2
Tuscany	1213	2	1
Tuscany	1225	1	1
Trentino-Alto Adige	2	1	1
Trentino-Alto Adige	32	1	1
Trentino-Alto Adige	40	3	3
Trentino-Alto Adige	100	1	1
Trentino-Alto Adige	106	1	1
Trentino-Alto Adige	121	1	1
Trentino-Alto Adige	124	4	4
Trentino-Alto Adige	149	6	6
Trentino-Alto Adige	153	3	3
Trentino-Alto Adige	165	5	3
Trentino-Alto Adige	190	2	2
Trentino-Alto Adige	191	3	2
Trentino-Alto Adige	192	5	3
Trentino-Alto Adige	221	10	6
Trentino-Alto Adige	258	3	2
Trentino-Alto Adige	282	1	1
Trentino-Alto Adige	295	3	3

Trentino-Alto Adige	343	2	2
Trentino-Alto Adige	359	1	1
Trentino-Alto Adige	365	4	3
Trentino-Alto Adige	370	1	1
Trentino-Alto Adige	373	3	2
Trentino-Alto Adige	439	9	4
Trentino-Alto Adige	450	1	1
Trentino-Alto Adige	466	2	1
Trentino-Alto Adige	491	1	1
Trentino-Alto Adige	503	1	1
Trentino-Alto Adige	531	1	1
Trentino-Alto Adige	567	7	5
Trentino-Alto Adige	586	1	1
Trentino-Alto Adige	601	3	1
Trentino-Alto Adige	674	1	1
Trentino-Alto Adige	713	2	1
Trentino-Alto Adige	719	5	4
Trentino-Alto Adige	770	7	4
Trentino-Alto Adige	800	1	1
Trentino-Alto Adige	802	2	2
Trentino-Alto Adige	879	1	1
Trentino-Alto Adige	931	2	2
Trentino-Alto Adige	982	3	2
Trentino-Alto Adige	999	1	1
Trentino-Alto Adige	1001	1	1
Trentino-Alto Adige	1011	3	2
Trentino-Alto Adige	1030	1	1
Trentino-Alto Adige	1063	2	2
Trentino-Alto Adige	1083	1	1
Trentino-Alto Adige	1088	2	1
Trentino-Alto Adige	1110	1	1
Trentino-Alto Adige	1130	3	2
Trentino-Alto Adige	1133	1	1
Trentino-Alto Adige	1135	1	1
Trentino-Alto Adige	1140	1	1
Trentino-Alto Adige	1173	1	1
Trentino-Alto Adige	1185	1	1
Trentino-Alto Adige	1201	1	1
Trentino-Alto Adige	1210	1	1
Trentino-Alto Adige	1214	1	1
Trentino-Alto Adige	1234	1	1
Trentino-Alto Adige	1236	2	1
Trentino-Alto Adige	1241	2	1
Trentino-Alto Adige	1243	2	1
Trentino-Alto Adige	1244	1	1
Umbria	49	1	1
Umbria	120	3	3
Umbria	169	1	1
Umbria	187	9	5
Umbria	207	1	1

Umbria	237	4	4
Umbria	272	1	1
Umbria	325	1	1
Umbria	399	5	5
Umbria	422	6	4
Umbria	426	1	1
Umbria	445	1	1
Umbria	447	1	1
Umbria	448	1	1
Umbria	464	3	3
Umbria	523	1	1
Umbria	531	6	4
Umbria	563	1	1
Umbria	575	1	1
Umbria	596	2	2
Umbria	662	1	1
Umbria	682	1	1
Umbria	784	1	1
Umbria	797	1	1
Umbria	849	1	1
Umbria	887	2	2
Umbria	916	3	2
Umbria	968	1	1
Umbria	1191	1	1
Umbria	1209	1	1
Umbria	1215	2	1
Umbria	1228	1	1
Valle d'Aosta	44	6	2
Valle d'Aosta	48	1	1
Valle d'Aosta	156	24	6
Valle d'Aosta	226	4	3
Valle d'Aosta	324	8	5
Valle d'Aosta	452	1	1
Valle d'Aosta	457	4	3
Valle d'Aosta	508	1	1
Valle d'Aosta	581	1	1
Valle d'Aosta	612	1	1
Valle d'Aosta	615	2	2
Valle d'Aosta	679	12	4
Valle d'Aosta	742	11	4
Valle d'Aosta	810	1	1
Valle d'Aosta	822	2	1
Valle d'Aosta	835	1	1
Valle d'Aosta	981	4	1
Valle d'Aosta	1075	1	1
Valle d'Aosta	1123	1	1
Valle d'Aosta	1193	1	1
Valle d'Aosta	1208	1	1
Valle d'Aosta	1238	1	1
Valle d'Aosta	1239	1	1

Veneto	18	1	1
Veneto	47	2	2
Veneto	52	13	5
Veneto	56	1	1
Veneto	57	2	2
Veneto	60	1	1
Veneto	61	1	1
Veneto	100	9	5
Veneto	115	3	2
Veneto	118	1	1
Veneto	123	4	3
Veneto	138	4	3
Veneto	167	8	5
Veneto	220	4	4
Veneto	224	1	1
Veneto	227	5	5
Veneto	233	6	4
Veneto	235	2	2
Veneto	240	1	1
Veneto	250	1	1
Veneto	276	12	6
Veneto	282	1	1
Veneto	292	1	1
Veneto	303	2	2
Veneto	309	1	1
Veneto	310	1	1
Veneto	311	1	1
Veneto	362	1	1
Veneto	364	1	1
Veneto	365	2	2
Veneto	369	2	2
Veneto	372	3	3
Veneto	374	2	2
Veneto	384	4	2
Veneto	408	3	3
Veneto	414	1	1
Veneto	415	2	2
Veneto	428	3	3
Veneto	431	2	2
Veneto	453	1	1
Veneto	479	1	1
Veneto	511	1	1
Veneto	513	3	2
Veneto	518	1	1
Veneto	520	9	3
Veneto	528	1	1
Veneto	550	8	4
Veneto	625	10	4
Veneto	648	4	2
Veneto	651	3	1

Veneto	670	1	1
Veneto	713	2	2
Veneto	715	1	1
Veneto	723	4	2
Veneto	731	3	1
Veneto	740	5	3
Veneto	744	18	4
Veneto	758	7	2
Veneto	764	1	1
Veneto	777	10	3
Veneto	799	4	2
Veneto	814	7	3
Veneto	832	3	1
Veneto	848	2	1
Veneto	858	4	2
Veneto	918	1	1
Veneto	931	3	3
Veneto	934	1	1
Veneto	942	1	1
Veneto	975	1	1
Veneto	990	2	2
Veneto	1012	1	1
Veneto	1014	1	1
Veneto	1021	2	2
Veneto	1038	1	1
Veneto	1041	1	1
Veneto	1042	1	1
Veneto	1056	1	1
Veneto	1070	1	1
Veneto	1073	1	1
Veneto	1074	1	1
Veneto	1076	1	1
Veneto	1081	1	1
Veneto	1104	1	1
Veneto	1120	1	1
Veneto	1128	2	2
Veneto	1130	1	1
Veneto	1143	1	1
Veneto	1166	1	1
Veneto	1181	1	1
Veneto	1190	1	1
Veneto	1194	1	1
Veneto	1195	1	1
Veneto	1201	1	1
Veneto	1206	1	1
Veneto	1207	1	1
Veneto	1216	2	1
Veneto	1237	3	1

Supplementary Table S7. Honey samples produced from 1986 to 2017.

Sample ID	Year	Region
1	1986	Emilia-Romagna
28	1987	Emilia-Romagna
34	1987	Emilia-Romagna
37	1988	Emilia-Romagna
44	1988	Emilia-Romagna
62	1988	Emilia-Romagna
30	1989	Emilia-Romagna
32	1989	Emilia-Romagna
33	1989	Emilia-Romagna
63	1989	Emilia-Romagna
29	1990	Emilia-Romagna
64	1990	Emilia-Romagna
27	1991	Emilia-Romagna
35	1991	Emilia-Romagna
55	1991	Emilia-Romagna
18	1992	Emilia-Romagna
66	1992	Emilia-Romagna
7	1993	Emilia-Romagna
43	1993	Emilia-Romagna
52	1993	Emilia-Romagna
53	1993	Emilia-Romagna
58	1993	Emilia-Romagna
11	1994	Emilia-Romagna
13	1994	Emilia-Romagna
20	1994	Emilia-Romagna
40	1994	Emilia-Romagna
8	1995	Emilia-Romagna
12	1995	Emilia-Romagna
14	1995	Emilia-Romagna
17	1995	Emilia-Romagna
36	1995	Emilia-Romagna
49	1995	Emilia-Romagna
6	1996	Emilia-Romagna
16	1996	Emilia-Romagna
50	1996	Emilia-Romagna
51	1996	Emilia-Romagna
42	1997	Emilia-Romagna
45	1997	Emilia-Romagna
48	1997	Emilia-Romagna
54	1997	Emilia-Romagna
47	1998	Emilia-Romagna
19	2000	Emilia-Romagna
23	2000	Emilia-Romagna

31	2000	Emilia-Romagna
39	2000	Emilia-Romagna
57	2000	Emilia-Romagna
65	2000	Emilia-Romagna
24	2001	Emilia-Romagna
38	2001	Emilia-Romagna
41	2001	Emilia-Romagna
61	2001	Emilia-Romagna
10	2002	Emilia-Romagna
21	2002	Emilia-Romagna
26	2002	Emilia-Romagna
4	2003	Emilia-Romagna
60	2003	Emilia-Romagna
2	2004	Emilia-Romagna
3	2004	Emilia-Romagna
5	2004	Emilia-Romagna
9	2004	Emilia-Romagna
25	2004	Emilia-Romagna
3	2005	Lombardia
8	2005	Lombardia
15	2005	Emilia-Romagna
46	2005	Emilia-Romagna
59	2005	Emilia-Romagna
1	2006	Piedmont
2	2006	Puglia
5	2006	Trentino-Alto Adige
6	2006	Lombardy
10	2006	Campania
16	2008	Umbria
146	2012	Piedmont
56	2012	Emilia-Romagna
41	2013	Emilia-Romagna
144	2013	Sicily
34	2014	Sicily
49	2014	Emilia-Romagna
50	2014	Sardinia
149	2014	Emilia-Romagna
157	2014	Sardinia
1	2015	Trentino-Alto Adige
28	2015	Emilia-Romagna
35	2015	Tuscany
36	2015	Friuli-Venezia Giulia
37	2015	Piedmont
40	2015	Tuscany
42	2015	Emilia-Romagna
43	2015	Umbria
46	2015	Sardinia

52	2015	Sicily
67	2015	Emilia-Romagna
88	2015	Emilia-Romagna
7	2016	Veneto
18	2016	Sardinia
27	2016	Sardinia
33	2016	Veneto
44	2016	Trentino-Alto Adige
45	2016	Emilia-Romagna
51	2016	Sardinia
53	2016	Lombardy
54	2016	Emilia-Romagna
59	2016	Emilia-Romagna
60	2016	Sardinia
112	2016	Tuscany
115	2016	Puglia
2	2017	Sardinia
4	2017	Piedmont
5	2017	Sicily
8	2017	Sicily
9	2017	Veneto
11	2017	Sardinia
12	2017	Sicily
13	2017	Abruzzo
14	2017	Abruzzo
15	2017	Abruzzo
16	2017	Tuscany
17	2017	Sicily
22	2017	Abruzzo
24	2017	Sicily
55	2017	Sicily
65	2017	Piedmont
69	2017	Valle d'Aosta
70	2017	Valle d'Aosta
71	2017	Abruzzo
72	2017	Calabria
74	2017	Abruzzo
76	2017	Sardinia
111	2017	Trentino-Alto Adige
127	2017	Friuli-Venezia Giulia
129	2017	Friuli-Venezia Giulia
130	2017	Friuli-Venezia Giulia
132	2017	Trentino-Alto Adige
135	2017	Calabria
136	2017	Campania
137	2017	Sardinia
138	2017	Basilicata
139	2017	Veneto
140	2017	Valle d'Aosta
141	2017	Calabria

142	2017	Calabria
143	2017	Veneto

Supplementary Figure S1. Density maps of the corresponding honey mtDNA profiles. **(a)** Honey samples with mitotype C. **(b)** Honey samples with mitotype A. **(c)** Honey samples with mitotype M.

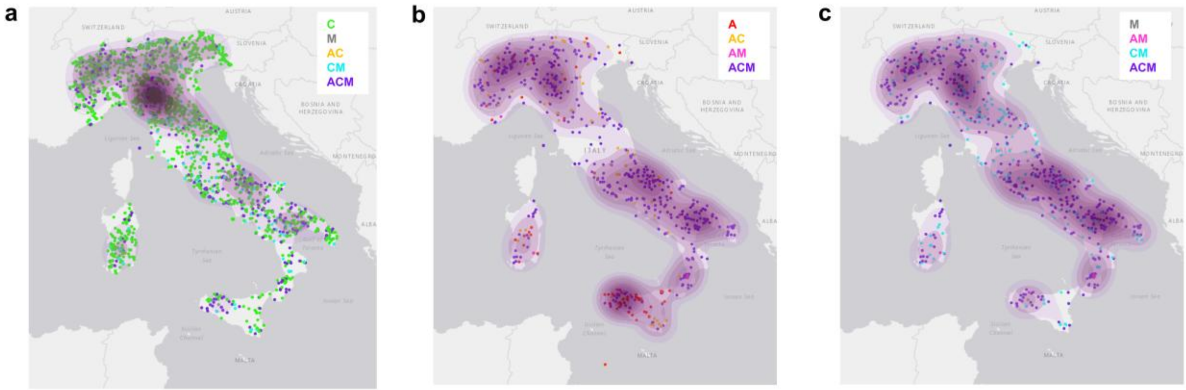

Supplementary Figure S2. Alignment of the targeted *Apis mellifera* mitochondrial DNA (mtDNA) region with the gaps indicated with "-" that can discriminate three main lineages [53]: A (152 bp), M (138 bp) and C (85 bp). Reported sequences are from the A1 mitotype (Gen-Bank/EMBL accession number: EF033649), the M4 mitotype (FJ743637) and C1 mitotype (FJ478010). Other mitotypes of the same lineages might differ on size for a few nucleotides. More details are reported in Utzeri et al. [53], which includes an extended alignment of *A. mellifera* mtDNA sequences. The PCR primer regions are underlined.

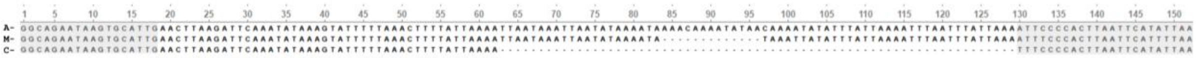

Supplementary Figure S3. Examples of gel electrophoresis patterns of the *Apis mellifera* mtDNA amplified fragments obtained from the DNA of several honey samples (lanes from 1 to 5): A lineage (band of 152 bp), M lineage (band of 138 bp) and C lineage (band of 85 bp). L: DNA ladder. Some honey samples had more than one band as described in the main text.

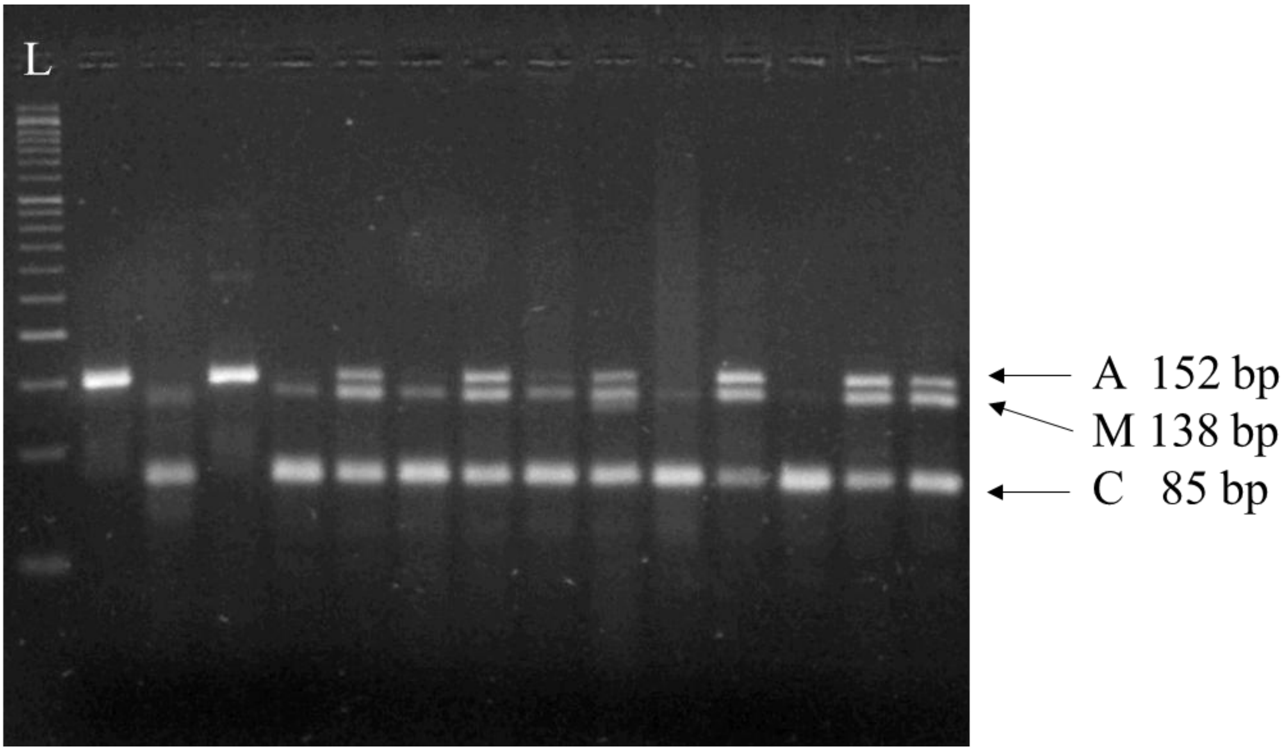

Supplement: Supplementary file 1 — Supplementary Material 1 [file 41598_2026_43936_MOESM1_ESM.pdf]
